# Insulin-Related Disordered Eating Behaviour: A Scoping Review of Evidence

**DOI:** 10.1007/s11892-026-01637-2

**Published:** 2026-07-29

**Authors:** Amy Shelford, Paul E. Jenkins, Kate Harvey

**Affiliations:** School of Psychology and Clinical Language Sciences, University of Reading, Whiteknights Campus, RG6 6ES Reading, England

**Keywords:** Type 1 diabetes, insulin-related disordered eating behaviour, insulin restriction, insulin inflation

## Abstract

**Purpose of Review:**

Current understanding of insulin-related disordered eating behaviour is limited by inconsistencies in terminology and measurement. Prevalence of the behaviour and characteristics of individuals are unclear. How best to intervene and with whom is therefore unknown. This scoping review summarises existing knowledge as a first step to addressing the problem. Eligible sources investigated individuals with T1D restricting or inflating insulin with disordered eating motivations. Charted data included study aims, sample characteristics, measures, and key findings.

**Recent Findings:**

Samples converged on adolescent females in USA, Canada and western Europe. Numerous measures were used, few had received extensive validation. Prevalence estimates, and models of development and maintenance were inconsistent.

**Summary:**

The validity and generalisability of measures should be improved, particularly so that diverse perspectives (e.g., males, non-Western cultures), so far overlooked, are captured. Identity and social relationships appeared particularly important in the development and maintenance of insulin-related disordered eating behaviour and recovery, and could be a potential target for interventions.

**Supplementary Information:**

The online version contains supplementary material available at 10.1007/s11892-026-01637-2.

## Introduction

Disordered eating is prevalent in adolescents and young adults with type 1 diabetes (T1D) [1, 2]. Insulin-related disordered eating behaviour (IRDEB) is unique to those managing their diabetes with insulin, mainly T1D, and includes both inflation and restriction of insulin doses [3–5].

Total or partial insulin restriction can be used to maintain increased glucose levels, inducing weight loss through similar mechanisms to those of pre-diagnosis and has been demonstrated as a separate construct from insulin restriction for other reasons [4, 6]. Insulin inflation is included in a novel operationalisation of IRDEB for the purpose of this review. Though documented, and anticipated as similarly prevalent as insulin omission [7], the mechanisms of insulin inflation as a disordered eating behaviour are poorly understood and under-researched [8, 9]. It therefore constitutes an emerging construct requiring further research.

Conclusions are difficult to draw due to: inconsistencies in terminology, screening, little knowledge about treatment needs, and minimal inquiry into insulin inflation generally [3, 6, 10, 11]. A comprehensive review of all available literature will help identify the potential impacts of IRDEB, its prevalence, and how it presents in individuals with T1D [12]. In light of the evolving understanding of disordered eating in diabetes, a synthesis of evidence is needed to clarify what is known in the area, identify evidence gaps and guide future research. These insights can also guide treatment efforts in routine diabetes care and aid understanding in clinicians who may be seeing such presentations more frequently [13]. Clarification of common concepts both between and across restrictive and inflation behaviours is necessary to determine whether a holistic or branched approach to research and treatment of IRDEB is more suitable. To achieve these requirements, a scoping review is the appropriate method of enquiry [14, 15].

The objective of this scoping review is to assess the extent of the literature regarding the IRDEB, encompassing insulin restriction and inflation, in people with T1D.

### Review Questions and Objectives

This scoping review aims to identify and summarise existing knowledge about IRDEB in T1D in order to address the following primary questions:

1. What is the nature of IRDEB, including prevalence, characteristics, and risk factors?
2. What gaps currently exist in the literature available on IRDEB?

Additional sub-questions to clarify consensus in the field include:

3. How is IRDEB screened for or measured?

## Methods

### Protocol and Registration

The review was designed and conducted in accordance with the Joanna Briggs Institute methodology for Scoping Reviews using the Preferred Items for Systematic Reviews and Meta-Analyses extension for Scoping Reviews [16, 17]. The protocol was preregistered on the Open Science Framework on 8 December 2023 [18],

### Eligibility Criteria

The Population Concept Context framework was used to define the eligibility criteria. Eligible evidence concerned individuals with T1D (population) either restricting or inflating insulin (concept), for the purpose of controlling weight or calorie absorption (context) [15]. Exclusions were applied to other types of diabetes due to the focus on insulin as the manipulated medication and the differences in mechanisms regarding the context of interest [19]. Exclusions also applied to alternative motives for insulin manipulation, including general non-adherence, self-harm and suicide. As a broad scoping review, all primary sources of evidence were included with no limits on date or language of publication, alongside grey literature, conference proceedings, and protocol registrations. Exclusions applied to news articles, and books and book chapters.

### Information Sources

Final searches were conducted on 24th November 2024 in PubMed, Scopus, Web of Science, ProQuest, and PsycINFO databases; Cochrane Reviews, Cochrane Trials, Open Science Framework Preprints, Open Science Framework Pre-registrations, and PROSPERO registers; and the Diabulimia Helpline website. Reference lists of included papers were hand-searched for additional evidence to be screened at the full-text level. The complete search strategy is detailed in the preregistration [18].

**Fig. 1:**
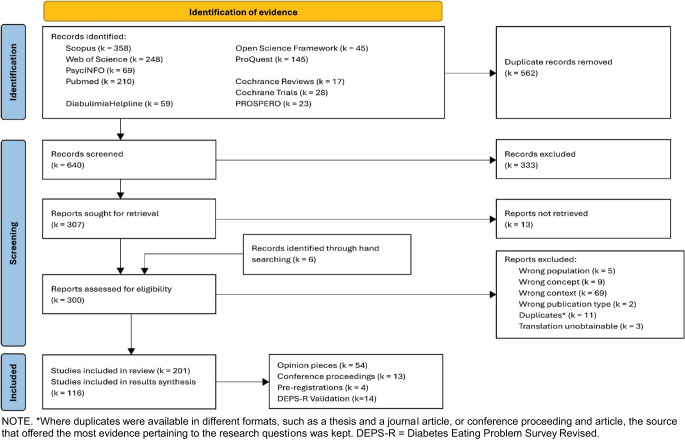
PRISMA flow diagram^1^

All titles and abstracts, and 10% full texts, were blindly double-screened with conflicts resolved through discussion. Exclusion reasons were recorded at full-text screening and detailed in the PRISMA diagram (Fig. 1). Screening instructions are detailed in the preregistration document [18, 20].

### Data Charting

A pilot data charting phase was completed with all design types, and subsequent data charting completed independently by AS. Data charting forms are included in the preregistration [18].

Opinion pieces including narrative reviews and editorials, pre-registrations and conference proceedings were included in the evidence base and organised thematically by aim. Studies reporting analysis of secondary data were excluded.

Data extracted from empirical evidence, Systematic and Scoping Reviews included country, aims, terminology, design, participant demographics, key findings, contributions to the area, and future research suggestions. Key findings represented results which most aligned with the purpose of this review rather than those highlighted by the individual study authors. Contributions to the area were taken as conclusions relative to each study’s findings on IRDEB.

### Critical Appraisal

Critical appraisals of primary research were performed using the Quality Assessment for Diverse Studies and can be found in Online Resource 1 [21].

### Differences from Original Registration

The pre-registration was updated twice during the pilot screening and data extraction phases to reflect two key changes: (1) All titles and abstracts were blindly double-screened to enhance consistency; and (2) opinion pieces (e.g., letters, editorials) and conference proceedings and pre-registrations were only categorised by topic and no further data were extracted.

### Synthesis of Results

Evidence was described relating to each review question and discussed relative to omission and restriction or inflation behaviours. This included describing the characteristics of the study samples; synthesis of prevalence of insulin manipulation; description of screening tools and terminology used. As an emerging construct with more limited evidence base, conclusions on insulin inflation should be interpreted with more caution than that of insulin restriction. Key proposed risk factors are described in Online Resource 2. Some evidence included in this review used the terms gender and sex interchangeably; this review will use the term sex for consistency. To describe the evidence base, key findings were arranged into themes to highlight areas of interest as suggested by Mak and Thomas [20], and suggestions for future research were synthesised to identify gaps and form recommendations.

## Results and Discussion

Given the number and variety of the sources identified, this section is divided into a description of the characteristics of the identified sources, a summary of which can be found in Online Resource 3, followed by a narrative synthesis. The terminology used across sources included in this review was inconsistent; this is considered in another paper [3].

### Description of Identified Sources

Figure 1 details the identification and screening process. Of the 201 sources included in the review, 116 were included in the results synthesis. References for all included sources can be found in Online Resource 4.

### Types and Focus of Identified Sources

Studies ranged from 1983 to the date of the search and included primary and secondary research. Table 1 details an overview of concepts across evidence, presented by focus and type of source.

**Table 1:** Aggregated overview of concepts, focus and types of sources identified

Focus	Type of source	Studies (*n*)	Overview of concept or aims
Insulin Inflation	Case reports	3^a^	• Controlling urges towards binge eating • Obtaining additional carbohydrates
	Systematic review	1	• Investigating motivations
Insulin Restriction & Inflation	Case reports	3^a^	• Psychopathology and social environment • Acute physiological consequences
	Model development	2	• Risk and maintenance of disordered eating in T1D • Qualitative data to inform treatment using CBT
	Protocol	1	• Proposed observational study to investigate progression and characteristics of disordered eating in T1D
	Quantitative	4	• A patient-led survey study exploring T1D care behaviours • Two studies investigate prevalence and motivations including disordered eating • Relating behaviours to eating disorder psychopathology
Insulin Restriction	Case reports	12^a^	• Conceptualisation as a disordered eating behaviour • Antecedents of behaviour development regarding diabetes care and social/family environment • Mechanisms in automatic closed loop systems • Physiological consequences • Psychological treatment and recommendations for approaches to healthcare
	Conference abstracts	13	• Tools to be used in measuring and screening • Approaches to and reviews of care pilots • Psychosocial antecedents and outcomes • Risk factors
	Model development	2	• Risk factor models consolidated from literature review
	Opinion	54	• Aetiology and relationship between disordered eating and T1D: prevalence, risk factors, and detection • Recommendations for care: treatment approaches, development of new care protocols, and methods and challenges of screening.
	Protocol	3	• Systematic review protocols for risk factors and prevalence • Intervention protocol for CBT and diabetes education
	Qualitative	12	• Experiences of individuals surrounding development of disordered eating and recovery factors • Parent experiences and influence of familial eating patterns • Perspectives of healthcare professionals • Parent and patient experiences of healthcare services
	Longitudinal	8^b^	• Demographic differences in severity • 5-, 10- and 14-year studies looked at development, prevalence, prognosis and mortality of disordered eating behaviours and “insulin restriction for weight control” • Comparison with non-T1D peers
	Quantitative	67	• Cross-sectional prevalence studies • Prognosis and physiological consequences • Nation-wide survey data to establish prevalence in USA, Germany, Belgium, Italy and Norway • Development of psychiatric, psychosocial and diabetes management profiles of individuals at risk or engaging in behaviours • Screening tools and measure development and validation • Treatment outcomes and recommendations for healthcare professionals • Intervention targeting insulin omission for weight loss with psychoeducation
	Meta-analysis	3	• Prevalence and risk factors associated with eating disorders or insulin restriction in T1D
	Systematic Review	4	• Evidence of use for weight control in males • Maintenance model of disordered eating in T1D • Influence of diabetes technology in disordered eating behaviour • Efficacy of interventions for insulin restriction • Recommendations for healthcare professionals
	Scoping Review	2	• Evidence of behaviour in males • Role of dieticians in care for patients

All references can be found in Online Resource 4; (a) Case reports constituted 12 publications; (b) longitudinal studies constituted 15 publications

### Methods Used in Identified Sources

#### Sample Characteristics

Characteristics of the 17,248 participants included in this review were taken from 18 case studies, 11 qualitative papers, and 67 quantitative papers. Samples were typically recruited from specialist diabetes clinics and ranged from single case designs to population-based studies of up to *n* = 2156. Across studies, the majority of participants were female (*n* = 10,150, 58.8%), with 41 of 90 (45.6%) studies (k = 10 case studies) recruiting female-only samples. In around half of studies (53%, *k* = 44), participants’ mean age was under 18 years, in nearly one-third participants’ mean age was 18–29 years (30%, *k* = 25) and in those remaining, participants’ mean age was over 30 years (17%, *k* = 14). Median duration of T1D was 7.6 years across all studies (IQR 6.4-12.8y). Across 54 studies reporting glycaemic control, 53 studies reported average HbA1c over the care target of 58mmol/mol (7.5%; [22]). In 56 papers recording ethnicity (67%), a White majority is reported.

#### Measures

Measures used to identify insulin-related disordered eating behaviours are described across 87 publications and eight conference proceedings, describing case studies, qualitative and quantitative studies. Four sources did not provide detail about the tools used [23–26].

Two case studies investigated only insulin inflation, using semi-structured interviews to identify the presence of and motivations for the behaviour [27, 28]. Seven studies measured both insulin omission and insulin inflation, three using custom self-report surveys [29–31], three using semi-structured interviews [7, 32, 33], and one a chart review of medical information [34]. Nine case studies documented insulin restriction using medical information from chart reviews (k = 5), semi-structured interviews (k = 4), and verbal disclosure as part of purposive sampling for the study (k = 4).

Twenty-seven studies used interviews, 11 of which were adaptations of validated interviews, most frequently the child or adult versions of the Eating Disorder Examination [35, 36]. Nine of the 27 used interviews in conjunction with other measures, such as questionnaires.

Forty-six studies used self-report questionnaire surveys: 12 were adaptations of existing validated surveys such as the Eating Disorder Inventory [37]; 19 were custom-created surveys, including one that was piloted before use [38], and two were created for previous studies [39, 40] and used by other research groups [41, 42].

Twenty-two studies used one of two instruments created to screen for disordered eating in diabetes: the Diabetes Eating Problems Survey – Revised (DEPS-R; [43]) was used in combination with other tools in five instances [43–47], and the modified SCOFF tool (mSCOFF; [48]) used in four studies [49–52]. A new screening questionnaire is currently being developed by the comPASSION project team [53].

#### Quantifying Insulin Restriction

A range of approaches was used to quantify insulin restriction: 10 studies quantified the timeframe for assessment, nine defined a threshold for the frequency of insulin restriction, four studies set a threshold and a timeline. Regarding frequency, discrete-choice scale studies commonly compiled answers indicating “at least sometimes” (e.g. [54]), or dichotomised options into “never” and “not never” (e.g. [6]), . Timeframes imposed on the measurement ranged from the preceding 24 h to the preceding 6 months. One study counted days of insulin restriction within the proposed timeframe [45]. Three studies set a threshold of restriction of dosage prescriptions by “at least ¼ prescribed insulin dose in a week” [55–57].

### Results of Identified Sources

In this section the findings of the sources included are synthesised to address the nature of insulin-related disordered eating including prevalence and characteristics. Due to the broad inclusion of study types, systematic reviews overlap with individual studies that are included. To avoid double-counting findings, when discussing prevalence only individual studies are considered, whereas when providing a synthesis of findings across sources, it is findings from systematic reviews that are considered, preferring those with more studies in cases of conceptual overlap (e.g. [58]., vs. [59]).

### Prevalence

Thirty-three of 64 quantitative papers (51.6%) measured the prevalence of insulin-related disordered eating as an aim, two of which included insulin inflation alongside insulin restriction. Prevalence statistics are synthesised from the variables indicated by authors as representative of insulin manipulation across 48 quantitative studies and baseline measurements from seven longitudinal studies. Prevalence statistics for all sources can be found in Online Resource 5.

#### Prevalence of Insulin Restriction

In samples diagnosed with eating disorders (k = 6), insulin restriction ranged from 13.2% [23] to 95.9% [60]. In non-clinical samples, 30 studies used surveys to measure insulin manipulation. Table 2 shows the range of prevalence rates for different orientations of motivation for omission and/or restriction of insulin.

**Table 2:** Orientations of questions measuring insulin omission and restriction using surveys with associated reported prevalence rates in non-clinical samples

Question orientation	Number of studies	Lowest prevalence rate	Highest prevalence rate
To control weight or shape	16	1% restricting overall 2.2% F, 0% M 1.4% omitting overall 1.5% F, 1.3% M 61	41% overall 92.3% F [29], 6.4% M [31]
In response to overeating or as a method of purging	5	25.8% restricting [62] 6.9% omitting overall [63]	61.5% restricting 61.2% F, 61.7% M 21.5% omitting 20.4% F, 22.5% M [64]
Defined by participant as weight-related	2	3.4% overall [30]	38% overall [42]
None	7	1.5% overall [65] 11.8% F, 0% M [19]	39.0% overall 37.7% F, 39% M [49]

*F* female, *M* male. Prevalence statistics for all sources in Online Resource 5

In eleven studies using interviews to measure insulin restriction, overall prevalence of insulin omission and/or restriction ranged between 3.7 and 35.7% [7, 66]. This ranged from 11 to 35.7% in females [66, 67], and 0-5.8% in males [68, 69].

#### Prevalence of Insulin Inflation

In three studies measuring insulin inflation (k = 4), motivations were qualitatively assigned by the participants, with weight control as a multiple-choice option in the fourth [56, 44, 18, 22]. Prevalence of insulin inflation in order to eat more ranged from 11.3% to 18%, with one study reporting a prevalence of 10.5% for females and 6.4% for males [31]. By contrast, in the only two papers describing case studies of insulin inflation, the majority were male (two out of three cases; [27, 28]).

#### Insulin Management Type

Four studies separated participants by management type, suggesting that individuals using multiple daily injections had higher prevalence of insulin-related disordered eating (15-71.8%) than those using a pump (0-21.1%; [7, 30, 41, 70]).

Inconsistencies in prevalence may be partially attributed to the variability in the operationalisation and measurement of insulin-related disordered eating. For example, suggested prevalence differences between females and males may have been exaggerated by the under-inclusion of males in earlier studies (e.g. [71]).

### Thematic Synthesis

Themes identified from the charted findings comprise: Possible Predictors of Insulin-Related Disordered Eating, Social Relationships, Identity, Characteristic Attitudes and Behaviours, Engaging Healthcare Professionals. Where overlap between individual studies and systematic reviews exists, this section uses findings from the systematic reviews in the interest of building on rather than repeating previous contributions.

#### Possible Predictors of Insulin-Related Disordered Eating

A theme within existing studies concerns the identification of attributes correlated with insulin-related disordered eating, and in some cases these are suggested for investigation as risk factors, though further, high-quality research is needed.

Diabetes-related correlates of IRDEB (k = 12) include duration of T1D, insulin management type including use of other diabetes technologies, and the presence and potential associated risk of ‘carbohydrate counting’. Non-diabetes-related correlates (k = 10) include factors such as emotion regulation, significant life events, and body mass index (BMI). There is suggestion that some risk factors are dynamic and their influence is related to individual differences [72]. Some research suggests an impact of culture and ethnicity [44, 52]. However, ethnicity is not typically considered as an independent variable, with only five (k = 4 doctoral theses) studies investigating ethnicity as a covariate.

#### Social Relationships

Social relationships were identified as an important theme, both as positive and negative influences in the development of and recovery from insulin-related disordered eating.

Negative influences (k = 19) included family conflict around food and T1D management, bullying from peers, and negative media interactions such as those espousing unrealistic body standards and diets [73]. Unhelpful or intrusive management advice and communications about diet, weight, and glucose control from parents and healthcare professionals (HCPs) also contributed to this theme [12, 13, 74, 75].

Positive or protective social influences (k = 9) included regular family mealtimes and supportive T1D co-management. Sensitivity and validation from HCPs in discussions around weight and food and using a person-centred approach are suggested to positively impact engagement with recovery [72, 76]. Positive social relationships with others that have engaged in insulin restriction, as well as supportive family and friends, can be powerful recovery support through destigmatisation as well as alleviation of loneliness [77]. Social media was mentioned as a source of recovery support in in qualitative studies and further investigation of this and its potential negative aspects could be valuable (e.g. [42]).

#### Identity

Qualitative studies identified the importance of identity in the use of insulin restriction to control weight and shape (k = 5). This includes descriptions of a reluctance to accept a diagnosis of T1D, a failure to integrate the diagnosis identity and changes to life and body arising from diagnosis, as a precursor to insulin restriction [32]. Another facet of this theme is the importance of differentiating; individuals with T1D who restrict their insulin prefer to be distinguished from those who do not, and from those with clinically diagnosed eating disorders. The labels “diabulimic” and “diabulimia” were endorsed by individuals engaging in insulin restriction across multiple sources, suggesting two implications [78]. Firstly, the lack of distinction may contribute to the failure of conventional eating disorder treatments to reduce insulin restriction [11, 79]. Secondly, the inconsistency in terms used potentially invites conflict in that individuals’ preferred term is not being used, exacerbating previously identified conflict between individuals and HCPs. This further supports the need for consistent terminology. A set of terms have recently been proposed, with the intention of harmonising the evidence base and interactions between researchers, HCPs and patients [3]. Consistent terminology will be essential when developing diagnostic criteria and care pathways, such as the pilots currently underway in the southwest of England [80], further supported by recent independent investigation [81].

#### Characteristic Attitudes and Behaviours

A key theme identified from evidence is the attitudes and behaviours shared by individuals engaging in insulin-related disordered eating. This theme encompasses: attitudes and care practices related to T1D diagnosis and management; facets of insulin-related disordered eating behaviour; and attitudes and behaviours adjacent but related to insulin-related disordered eating.

T1D-related attitudes (k = 13) include illness perceptions and maladaptive attitudes towards insulin such that T1D is unmanageable, disruptive, or limiting, and that insulin is fattening, or a weight control tool Some individuals prioritise weight loss over effective diabetes management, which is reflected in disregard for other diabetes care practices such as monitoring blood glucose (k = 9).

Due to the varied ways in which constructs have been operationalised and measured across the evidence base, different active and passive behaviours within insulin-related disordered eating have been revealed [3]. Individuals may *restrict* insulin in the following ways: reducing a meal or snack bolus; reducing or omitting a bolus intended to correct a high glucose level; reducing or omitting a basal dose; eating to raise the glucose level without a bolus. Individuals may *inflate* their insulin in the following ways: increasing a bolus dose; administering an additional bolus dose; using the canular priming function on an insulin pump to administer an additional dose of insulin that is not recorded in the main memory.

A final facet of the theme includes evidence which illustrates the attitudes and behaviours adjacent to insulin-related disordered eating (k = 26). This includes high levels of eating psychopathology, poor body image, inappropriate weight control practices such as laxative misuse and extreme dietary restriction, skipping meals and binge eating [55].

#### Engaging Healthcare Professionals

The engagement of HCPs has become apparent in empirical research in the last decade (k = 8), establishing their experiences with individuals engaging in insulin-related disordered eating and piloting services and models for care. The services being developed are still in their infancy, and HCPs in both eating disorder services and diabetes care teams often report feeling ‘out of their depth’ with this comorbidity. This unease can be exacerbated by inconsistencies in terminology and operationalisation, making it difficult to find information [13], and can lead to unhelpful or misguided communications [78].

### Strengths and Limitations of Scoping Review

Strengths of this scoping review include the adherence to best practice and inclusion of grey literature which enabled a comprehensive view of available research, such as when a thesis was included over the associated journal article in favour of the detail afforded to the content of interest and the comprehensive nature of the review.

Limitations of this scoping review include the inability to obtain 13 papers shortlisted for full-text screening, despite contacting corresponding authors. Similarly, English-language translations of three full-text papers were unobtainable. This may have impacted the geographical distribution of research, however in such a large pool of evidence, it may not have had a significant impact on the conclusions of the review. The use of broad inclusion criteria resulted in a large base of evidence for synthesis, and less detailed consideration of opinion pieces. Consideration of opinion literature could have revealed how researchers in the area view the evidence base and guided the conclusions in terms of necessitated clarity.

### Contribution to Research

This scoping review contributes to our understanding of insulin-related disordered eating by identifying what is known and what has yet to be established. It will support future research by highlighting inconsistencies in the current evidence base, and suggesting gaps in knowledge that have been identified during the review process.

#### Directions for Future Research

Biases in the samples recruited limit the generalisability of findings beyond females, adolescents and adults under 30 years old, and those engaged with clinical services. This indicates a need for study recruitment to broaden beyond diabetes clinics and include a balance of male participants. Given the binary reporting of gender and sex across studies, future work might consider a more nuanced interpretation of gender, considering links with constructs such as identity, social relationships, eating behaviour, and body image (e.g. [82]), .

The wide variation in terminology, methods and tools used in the area invites caution in drawing conclusions across the evidence base, and warrants the development of a reliable tool for screening and measurement of IRDEB [3, 83]. An interview may afford clarity around the motivations for insulin manipulation as defined by the participant, and whether the behaviours align with disordered eating ascertained with greater confidence.

These aspects of intersectionality and accuracy of terminology and measurement should be addressed in the prospective longitudinal studies that the JDRF Parliamentary Report calls for [84]. Research on risk factors and models of development and maintenance should also be extended, considering intersectionality in the testing of existing models so that they can be used as a foundation for preventative and recovery strategies.

The evidence base for insulin inflation is limited to a high proportion of case studies and limited number of cohort studies. Future investigations should address this: aiming to understand and identify insulin inflation, and ascertain whether insulin inflation in this context needs to be separated from insulin restriction, or if their mechanisms are linked.

There is still qualitative investigation needed to deepen understanding of the development and maintenance of insulin-related disordered eating behaviours, whilst also addressing the male perspective. Preliminary differences in cultural investigation and demonstration of insulin-related disordered eating may highlight a valuable avenue to explore with qualitative ethnographic study.

Evidence relating to the recovery from insulin-related disordered eating is notably sparse. With an initial demonstration of the importance of social relationships in the development of insulin-related disordered eating, further research investigating the influence of HCP, family, and online and offline peer relationships in the maintenance and recovery is needed, as these could be valuable targets for intervention.

## Conclusions

This review aimed to synthesise evidence on the prevalence and characteristics associated with insulin-related disordered eating, and identify gaps and future directions for research in the area. Characteristics of samples converge on White adolescent females. Several tools are used to assess insulin-related disordered eating, but few have extensive validation. Prevalence estimates and evidence for risk factors for insulin-related disordered eating behaviour are inconsistent. It is therefore unsurprising that HCPs may feel uninformed and ill-prepared in providing care and support with individuals and parents, and would like to be trained in how to approach this. Facets of identity, and relationships with HCPs, friends and family, appear influential in the development and maintenance of insulin-related disordered eating behaviour: future research should investigate potential implications of this in recovery. To improve measures and generalisability, future research needs to establish and incorporate diverse perspectives, particularly those of males and non-western cultures.

## Key References

- Albaladejo L, Périner-Marquet P, Buis C, Lablanche S, Iceta S, Arnol N, et al. High prevalence with no gender difference of likely eating disorders in type 1 mellitus diabetes on insulin pump. DIABETES RESEARCH AND CLINICAL PRACTICE. 2023;199:110630. doi: 10.1016/j.diabres.2023.110630.
  - ○ This paper importantly challenges the assumptions that prevalence of insulin-related disordered eating behaviour is higher in females and adolescence, and that individuals will present with less than satisfactory diabetes management markers, such as high glucose, highlighting the need to give appropriate attention to higher age groups and male counterparts, as well as carefully considering means for identifying individuals with whom to intervene.
- Goddard G, Oxlad M. Insulin restriction or omission in Type 1 Diabetes Mellitus: a meta-synthesis of individuals' experiences of diabulimia. Health Psychology Review. 2023;17(2):227-46. doi: 10.1080/17437199.2021.2025133.
  - ○ This paper importantly synthesises all qualitative evidence on the experience of insulin restriction. In an area so flooded by quantitative research with inconsistent terminology and measurement of behaviours, this paper is paramount for the illumination of the patient experience and re-establishment of patient-centred research.
- Shelford A, Jenkins PE, Harvey K. Insulin-Related Disordered Eating Behaviour: Clarifying Terminology. International Journal of Eating Disorders. 2025. doi: 10.1002/eat.24548.
  - ○ This paper importantly discusses the impact of inconsistent terminology in an area with such adverse outcomes for individuals engaging in insulin-related disordered eating behaviour, and suggests umbrella terms to be used to harmonise evidence, research and practice.

## Electronic Supplementary Material

Below is the link to the electronic supplementary material.

Supplementary file 1

Supplementary file 2

Supplementary file 3

Supplementary file 4

Supplementary file 5

## Data Availability

The charted data generated during the current study is available on the Open Science Framework, osf.io/yu3tk, available here: [https://osf.io/6be9j/files/osfstorage].

## Footnotes

1 Figurepreviously published in [3]
