# Supplementary material for: Insulin-Related Disordered Eating Behaviour: A Scoping Review of Evidence
Source: Curr Diab Rep. 2026 Jul 29;26(1):23. doi: 10.1007/s11892-026-01637-2 (PMC13415336; doi:10.1007/s11892-026-01637-2)
Supplement: Supplementary file 1 [file 11892_2026_1637_MOESM1_ESM.pdf]

S1: Critical Appraisal of Identified Sources

There was variability among the primary research sources in terms of clarity and good practice. The systematic and scoping reviews included generally used PRISMA guidelines to describe their rigour, though one early meta-analysis did not use any guidelines or frameworks (Nielsen, 2002).

Critical appraisal of 87 primary studies was performed using the QUADS (R. Harrison et al., 2021). This tool was chosen because it can be applied across all study designs thus avoiding the need to use multiple quality appraisal tools. The QUADS does not provide a rating metric, as studies of different designs may not be comparable. Instead, it gives criteria by which to evaluate elements of research that are applicable across designs, such as the presence and strength of rationale, justification of methods used, and critical analysis. Criteria have been grouped according to different aspects of research (see Table 2) and will be described using these groupings.

Table S1. Definition and mapping of QuADS criteria onto reported groupings.

QuADS criterion identifier	Reporting group
1. Theoretical or conceptual underpinning to the research	Rationale, aims and design
2. Statement of research aim/s	Rationale, aims and design
3. Clear description of research setting and target population	Samples and recruitment
4. The study design is appropriate to address the stated research aim/s	Rationale, aims and design
5. Appropriate sampling to address the research aim/s	Samples and recruitment
6. Rationale for choice of data collection tool/s	Data collection
7. The format and content of data collection tool is appropriate to address the stated research aim/s	Data collection
8. Description of data collection procedure	Data collection
9. Recruitment data provided	Samples and recruitment
10. Justification for analytic method selected	Analysis and discussion
11. The method of analysis was appropriate to answer the research aim/s	Analysis and discussion
12. Evidence that the research stakeholders have been considered in research design or conduct.	Rationale, aims and design
13. Strengths and limitations critically discussed	Analysis and discussion

* akshelford@gmail.com

School of Psychology and Clinical Language Sciences, University of Reading, UK

Rationale, aims and design (criteria 1, 2, 4 & 12)

Few studies used models as a theoretical underpinning, though as an emerging area this criterion was mainly evaluated from the source's discussion of previous evidence and rationale for the study. Studies clearly outlined the research aims with few exceptions, and were appropriately designed to address their aims. Some included the aim of piloting custom measures.

Samples and recruitment (criteria 3, 5 & 9)

Description of the research setting was generally deemed to be poor, though most studies included eligibility criteria for recruitment. Many recruited from clinic samples, biasing evidence towards those who have maintained their clinic attendance. Justification for samples and sample size was scarce. Most studies included basic recruitment data (i.e., number agreeing to participate) with many including details of numbers invited as well as attrition data.

Data collection (criteria 6, 7 & 8)

Typically, there was no justification for the measures used, other than reference to use in a previous study; for example, Battaglia et al. (2006) added two questions to their custom-created survey that were used by Meltzer et al. (2001) to measure insulin omission. Measures were often unable to provide insight regarding the aims of insulin restriction as they were not designed to assess weight or shape control motivations (e.g., DEPS-R; mSCOFF). Most studies included a brief outline of data collection procedures, with some giving more detail for each stage.

Analysis and discussion (criteria 10, 11 & 13)

Studies described their analyses in the Methods sections in varying detail, ranging from stating the planned analysis to mapping measures onto variables. Analysis was deemed appropriate to the aims with few exceptions. Studies published before 2005 generally had less detailed critical discussions and often provided limited discussion of limitations or future directions. Some studies acknowledged the poor operationalisation of insulin manipulation measures in their critical discussion.

* akshelford@gmail.com

School of Psychology and Clinical Language Sciences, University of Reading, UK
