# Supplementary material for: Insulin-Related Disordered Eating Behaviour: A Scoping Review of Evidence
Source: Curr Diab Rep. 2026 Jul 29;26(1):23. doi: 10.1007/s11892-026-01637-2 (PMC13415336; doi:10.1007/s11892-026-01637-2)
Supplement: Supplementary file 2 [file 11892_2026_1637_MOESM2_ESM.pdf]

S2: Description of key risk factors discussed in included sources

Five studies have developed models of risk factors for insulin restriction (Beam et al., 2021; De Paoli & Rogers, 2018; Harrison et al., 2021; Peterson et al., 2015; Treasure et al., 2015), broadly identifying two types of risk factor: those relating the T1D and diagnosis; and those not. Non-T1D-related risk factors include sex, age, and more recently proposed emotion regulation and personality factors. T1D-related risk factors for insulin-related disordered eating include age of diagnosis, duration of T1D, diagnosis experience surrounding weight changes, and related interactions with HCPs and family. Evidence to test the existing models, including newly proposed risk factors such as emotionality and personality factors, is lacking, therefore this section is limited to the evidence on sex, age, and age of T1D diagnosis.

Sex

Opinion pieces claiming sex as a risk factor (e.g., Haagen, 2011) might be countered by the apparent female dominance (58.5%) observed across samples. Recently, samples more evenly distributed across sexes have suggested similar prevalence rates between males and females (e.g., Baechle et al., 2019). Thus, further research is needed to investigate the sensitivity and validity of measures in male samples, so that the role of biological sex as a factor in the development of insulin-related disordered eating can be determined (Eilander et al., 2017; Harrison et al., 2021).

Age

Age as a risk factor for insulin-related disordered eating was investigated in relation to the development of identity and the relation of this to eating disorder development in the general population (Haagen, 2011; Rancourt et al., 2019). A longitudinal prospective series of studies using semi-structured interviews observed insulin restriction for weight control throughout the ages of 9 to 26 years, with the highest incidence at the latest timepoint (ages 21-26 years) (P. Colton et al., 2007; Colton et al., 2004; Colton et al., 2015; P. A. Colton et al., 2007). Other evidence using the DEPS-R to measure insulin restriction finds no significant association with age (e.g. Troncone et al., 2022; Watt et al., 2022). The format of measures differed between these studies, and the DEPS-R questions are not oriented towards disordered eating motivations, which may result in a reduced sensitivity to detect age-related patterns. However, awareness of insulin-related disordered eating may be higher in the later studies following its integration into popular media, (e.g. *Diabulimia: The world's most dangerous eating disorder;* BBC 3, 2017). This suggests that whilst age may be relevant, there is a complex interplay of

* akshelford@gmail.com

School of Psychology and Clinical Language Sciences, University of Reading, UK

other factors relating to living with T1D that may be masked if age alone is investigated. This has led to the investigation of age of T1D diagnosis as a risk factor.

Age of T1D diagnosis

Some literature suggests that receiving a T1D diagnosis at an early age may influence the likelihood of engagement in subsequent insulin restriction (Bächle et al., 2016). Other evidence suggests a T1D diagnosis in adolescence disrupts identity development and increases likelihood of insulin restriction (Gottesman et al., 2015; Pinhas-Hamiel et al., 2013). The majority of research has been conducted in the adolescent age group, and so research across broader age groups may provide more clarity.

* akshelford@gmail.com

School of Psychology and Clinical Language Sciences, University of Reading, UK
