# Supplementary material for: Insulin-Related Disordered Eating Behaviour: A Scoping Review of Evidence
Source: Curr Diab Rep. 2026 Jul 29;26(1):23. doi: 10.1007/s11892-026-01637-2 (PMC13415336; doi:10.1007/s11892-026-01637-2)
Supplement: Supplementary file 3 [file 11892_2026_1637_MOESM3_ESM.pdf]

S3: Summary of Description of Identified Sources

Table S3. Table of main points per section of part one: description of identified sources

Section	Summary points
Types of Identified Sources	• Research spans from 1983 to present<br>• The majority of outputs in the field are quantitative primary sources (k=70) closely followed by opinion-based literature (k=54)<br>• From 2015 onwards, there is an increase in qualitative and systematic review outputs as well as conference proceedings
Focus of Identified Sources	• Three outputs focus on insulin overdose as a disordered eating behaviour, mainly represented in the western hemisphere<br>• Five outputs solely represented in the western hemisphere, mainly in the UK, investigate both insulin restriction and overdose as disordered eating behaviours<br>• All opinion pieces, conference abstracts, and the majority of primary research outputs related to insulin restriction, with the strongest representation in the USA, followed by the UK<br>• Primary research relating to insulin restriction also contains longitudinal study, and investigation of HCP perspectives
Methods Used in Identified Sources: Sample Characteristics	• Of 17,248 participants across primary outputs, 58.8% were female (n=10,150)<br>• In over half of the primary studies (53%), samples had a mean age under 18<br>• Ethnicity is recorded in 67% primary studies, describing a Caucasian majority
Methods Used in Identified Sources: Measures	• Measurement of insulin-related disordered eating includes a chart review of medical information, semi-structured interviews, and surveys<br>• Semi-structured interviews and surveys are evenly split in application in studies focusing on both insulin overdose and insulin restriction<br>• Semi-structured interviews may be custom-created, or adaptations of tools validated in the general population<br>• Semi-structured interviews are more likely to follow up on and establish motivations for insulin manipulation to confirm the behaviour as disordered eating<br>• Surveys may be custom-created, or adaptations of tools validated in the general population<br>• Surveys are more commonly used in studies focusing only on insulin restriction

* akshelford@gmail.com

School of Psychology and Clinical Language Sciences, University of Reading, UK

Section	Summary points
	• Surveys specifically created for screening of disordered eating in people with diabetes (DEPS-R, mSCOFF) do not anchor their questions with motivations for the behaviour of interest, and only concern insulin restriction<br>• Quantitative studies vary in their approach to quantifying insulin restriction in terms of frequency and timeline thresholds

Note: DEPS-R = Diabetes Eating Problem Survey – Revised (Markowitz et al., 2010); HCP = healthcare professional; mSCOFF = modified SCOFF (Zuijdwijk et al., 2014)

* akshelford@gmail.com

School of Psychology and Clinical Language Sciences, University of Reading, UK
