# Supplementary material for: Insulin-Related Disordered Eating Behaviour: A Scoping Review of Evidence
Source: Curr Diab Rep. 2026 Jul 29;26(1):23. doi: 10.1007/s11892-026-01637-2 (PMC13415336; doi:10.1007/s11892-026-01637-2)
Supplement: Supplementary file 4 [file 11892_2026_1637_MOESM4_ESM.pdf]

S4: references of all papers included in scoping review

*Denotes papers referenced in text

*Abild, C. B., Vestergaard, E. T., Bruun, J. M., Kristensen, K., Stoving, R. K., & Clausen, L. (2024). Mechanisms underlying the development of eating disorders and disordered eating in adolescent females with type 1 diabetes. Diabetic Medicine, 41(11), 11. https://doi.org/doi:10.1111/dme.15397

*Abraham, S. A., Aarella, V. G., & Fernandez, C. (2020). Diabulimia: A case report highlighting the importance of a multidisciplinary approach to the care of patients with eating disorders associated with type 1 diabetes. *Diabetic Medicine*, *37*, 77-77.

*Ackard, D. M., Vik, N., Neumark-Sztainer, D., Schmitz, K. H., Hannan, P., & Jacobs, D. R. (2008). Disordered eating and body dissatisfaction in adolescents with type 1 diabetes and a population-based comparison sample: comparative prevalence and clinical implications. *Pediatric Diabetes*, *9*(4), 312-319. https://doi.org/doi:10.1111/j.1399-5448.2008.00392.x

Affenito, S. A., Backstrand, J. R., Welch, G. W., Lammi-Keefe, C. J., Rodriguez, N. R., & Adams, C. H. (1997). Subclinical and Clinical Eating Disorders in IDDM Negatively Affect Metabolic Control. *Diabetes Care*, *20*(2), 182-184.

Affenito, S. G., & Adams, C. H. (2001). Are eating disorders more prevalent in females with type 1 diabetes mellitus when the impact of insulin omission is considered? *Nutrition Reviews*, *59*(6), 179-182. https://doi.org/doi:10.1111/j.1753-4887.2001.tb07010.x

Ahsan, Z. (2020). *Diabulimia: A Systematic Review of Treatment* Kansas City University of Medicine and Biosciences]. ProQuest Dissertation and Thesis. https://www.proquest.com/openview/8031e0f675eb12dbdc3371e142d911ac/1?cbl=18750&diss=y&pq-origsite=gscholar&forcedol=true

*Albaladejo, L., Périner-Marquet, P., Buis, C., Lablanche, S., Iceta, S., Arnol, N., Logerot, S., Borel, J. C., & Bétry, C. (2023). High prevalence with no gender difference of likely eating disorders in type 1 mellitus diabetes on insulin pump. *Diabetes Research and Clinical Practice*, *199*, 110630. https://doi.org/doi:10.1016/j.diabres.2023.110630

*Allan, J. A. (2015). Understanding poor outcomes in women with type 1 diabetes and eating disorders. *Journal of Diabetes Nursing*, *19*(3), 99-103.

*Araia, E., Hendrieckx, C., Skinner, T., Pouwer, F., Speight, J., & King, R. M. (2017). Gender differences in disordered eating behaviors and body dissatisfaction among adolescents with type 1 diabetes: Results from diabetes MILES youth-Australia. *Int J Eat Disord*, *50*(10), 1183-1193. https://doi.org/doi:10.1002/eat.22746

Atik Altınok, Y., Özgür, S., Meseri, R., Özen, S., Darcan, Ş., & Gökşen, D. (2017). Reliability and validity of the diabetes eating problem survey in Turkish children and adolescents with type 1 diabetes mellitus. *JCRPE Journal of Clinical Research in Pediatric Endocrinology*, *9*(4), 323-328. https://doi.org/doi:10.4274/jcrpe.4219

Atriham, A. R., Kleszczyński, J., Sierakowska, A., & Springer, J. (2024). Diabulimia – a diagnostic and therapeutic challenge in the Emergency Department. European Journal of Translational and Clinical Medicine, 7(1), 57-62. https://doi.org/doi:10.31373/ejtcm/183021

* akshelford@gmail.com

School of Psychology and Clinical Language Sciences, University of Reading, UK

*Bächle, C., Stahl-Pehe, A., & Rosenbauer, J. (2016). Disordered eating and insulin restriction in youths receiving intensified insulin treatment: Results from a nationwide population-based study. *International Journal of Eating Disorders*, *49*(2), 193-198. https://doi.org/10.1002/eat.22463

*Baechle, C., Castillo, K., Strassburger, K., Stahl-Pehe, A., Meissner, T., Holl, R. W., Giani, G., Rosenbauer, J., German Paediatric Surveillance, U., & the, D. P. V. S. I. (2014). Is disordered eating behavior more prevalent in adolescents with early-onset type 1 diabetes than in their representative peers? *Int J Eat Disord*, *47*(4), 342-352. https://doi.org/doi:10.1002/eat.22238

*Baechle, C., Hoyer, A., Stahl-Pehe, A., Castillo, K., Toennies, T., Lindner, L. M. E., Reinauer, C., Holl, R. W., Kuss, O., & Rosenbauer, J. (2019). Course of Disordered Eating Behavior in Young People With Early-Onset Type I Diabetes: Prevalence, Symptoms, and Transition Probabilities. *Journal of Adolescent Health*, *65*(5), 681-689. https://doi.org/doi:10.1016/j.jadohealth.2019.05.016

Balfe, M., Doyle, F., Smith, D., Sreenan, S., Conroy, R., & Brugha, R. (2013). Dealing with the devil: weight loss concerns in young adult women with type 1 diabetes. *Journal of Clinical Nursing*, *22*(13), 2030-2038. https://doi.org/doi:10.1111/jocn.12231

*Battaglia, M. R., Alemzadeh, R., Katte, H., Hall, P. L., & Perlmuter, L. C. (2006). Brief Report: Disordered Eating and Psychosocial Factors in Adolescent Females with Type 1 Diabetes Mellitus. *Journal of Pediatric Psychology*, *31*(6), 552-556. https://doi.org/doi:10.1093/jpepsy/jsj047

*Bauman, V., Sturkey, A., Sherafat-Kazemzadeh, R., McEwan, J., Jones, P., Keating, A., Isganaitis, E., Ricker, A., & Rother, K. (2018). Factitious hypoglycaemia in children and adolescents with diabetes. *Pediatr Diabetes*, *19*, 823-831. https://doi.org/doi:10.1111/pedi.12650

*Beam, A. B., Wiebe, D. J., & Berg, C. A. (2021). Insulin Restriction, Emotion Dysregulation, and Depressive Symptoms in Late Adolescents with Diabetes. *Journal of Pediatric Psychology*, *46*(9), 1110-1118. https://doi.org/10.1093/jpepsy/jsab042

Bermudez, O., Gallivan, H., Jahrus, J., Lesser, J., Meier, M., Parkin, C. (2009). Inpatient management of eating disorders in type 1 diabetes. *Diabetes Spectrum*, *22*(3), 153-158.

*Biggs, M., Ramirez Basco, M., Patterson, G., & Raskin, P. (1994). Insulin withholding for weight control in women with diabetes. *Diabetes Care*, *17*(10), 1186-1189. https://doi.org/doi:10.2337/diacare.17.10.1186

Bordner, C., Stuckey-Peyrot, H. L., Laffel, L. M., Prokop, A., Toschi, E., & Goebel-Fabbri, A. E. (2024, 2024-9-17). 1846-LB: Developing Best Practices for Preventing "Diabulimia"-Focusing on Health Care Professionals' Knowledge of Insulin Omission/Restriction. Diabetes, suppl. Supplement 1,

*Bowden, M. M., Eddington, A., Keenan, M., Berlin, K., Semenkovich, K., & Alemzadeh, R. (2018). Disordered Eating Behaviour and Insulin Omission in Type 1 Diabetes: mSCOFF as a Screening Tool. 2018 Southern Regional Meeting, Journal of Investigative Medicine,

*Brewster, S., Partridge, H., Cross, C., & Price, H. (2020). Healthcare professional awareness of eating disorders in people with type 1 diabetes: a staff survey. *BRITISH JOURNAL OF DIABETES*, *20*(2), 122-130. https://doi.org/doi:10.15277/bjd.2020.268

*Broadley, M. M., Zaremba, N., Andrew, B., Ismail, K., Treasure, J., White, M. J., & Stadler, M. (2020). 25 Years of psychological research investigating disordered eating in people with diabetes: what have we learnt? *Diabetic Medicine*, *37*(3), 401-408. https://doi.org/doi:10.1111/dme.14197

* akshelford@gmail.com

School of Psychology and Clinical Language Sciences, University of Reading, UK

*Bryden, K. S., Neil, A., Mayou, R. A., Peveler, R., Fairburn, C. G., & Dunger, D. B. (1999). Eating Habits, Body Weight, and Insulin Misuse. *Diabetes Care*, *22*(12), 1956-1960.

*Bubb, J. A., & Pontious, S. L. (1991). Weight Loss From Inappropriate Insulin Manipulation: An Eating Disorder Variant in an Adolescent With Insulin-Dependent Diabetes Mellitus [Article]. The Diabetes Educator, 17(1), 29-32. https://doi.org/10.1177/014572179101700106

Cainer, A. (2022). Recognising and managing type 1 disordered eating in children and young people with diabetes. *Nursing children and young people*, *34*(2), 28-32. https://doi.org/doi:10.7748/ncyp.2022.e1396

Callum, A. M., & Lewis, L. M. (2014). Diabulimia among adolescents and young adults with Type 1 diabetes. *Clinical Nursing Studies*, *2*(4). https://doi.org/10.5430/cns.v2n4p12

Cantwell, R., & Steel, J. M. (1996). Screening for Eating Disorders in Diabetes Mellitus. *Journal of Psychosomatic Research*, *40*(1), 15-20.

Cherubini, V., Skrami, E., Iannilli, A., Cesaretti, A., Paparusso, A. M., Alessandrelli, M. C., Carle, F., Ferrito, L., & Gesuita, R. (2018). Disordered eating behaviors in adolescents with type 1 diabetes: A cross-sectional population-based study in Italy. *International Journal of Eating Disorders*, *51*(8), 890-898. https://doi.org/doi:10.1002/eat.22889

*Chou, W. C., Chou, Y. Y., Pan, Y. W., Ou, T. Y., & Tsai, M. C. (2023). Correlates of disordered eating and insulin restriction behavior and its association with psychological health in Taiwanese youths with diabetes mellitus. *Journal of Eating Disorders*, *11*(1), 158. https://doi.org/doi:10.1186/s40337-023-00888-8

*Coleman, S. E., & Caswell, N. (2020). Diabetes and eating disorders: an exploration of 'Diabulimia'. *BMC Psychol*, *8*(1), 101. https://doi.org/10.1186/s40359-020-00468-4

*Colton, P. A., Olmsted, M. P., Daneman, D., Farquhar, J. C., Wong, H., Muskat, S., & Rodin, G. M. (2015). Eating Disorders in Girls and Women With Type 1 Diabetes: A Longitudinal Study of Prevalence, Onset, Remission, and Recurrence. *Diabetes Care*, *38*(7), 1212-1217. https://doi.org/10.2337/dc14-2646

*Colton, P. A., Olmsted, M. P., Daneman, D., Rydall, A. C., & Rodin, G. M. (2007). Five-year prevalence and persistence of disturbed eating behavior and eating disorders in girls with type 1 diabetes. *Diabetes Care*, *30*(11), 2861-2862. https://doi.org/doi:10.2337/dc07-1057

*Colton, P., Olmstead, M. P., Daneman, D., Rydall, A., & Rodin, G. (2007). Natural history and predictors of eating disturbances in girls with type 1 diabetes. *Diabetic Medicine*, *24*, 424-429. https://doi.org/DOI: 10.1111/j.1464-5491.2007.02099.x

*Colton, P., Rydall, A., Olmsted, M., Rodin, G., & Daneman, D. (2004). Disturbed Eating Behaviour and Eating Disorders in Preteen and Early Teenage Girls with Type 1 Diabetes. *Diabetes Care*, *27*(7), 1654-1659.

*Custal, N., Arcelus, J., Aguera, Z., Bove, F. I., Wales, J., Granero, R., Jimenez-Murcia, S., Sanchez, I., Riesco, N., Alonso, P., Crespo, J. M., Virgili, N., Menchon, J. M., & Fernandez-Aranda, F. (2014). Treatment outcome of patients with comorbid type 1 diabetes and eating disorders. *BMC Psychiatry*, *14*(140).

* akshelford@gmail.com

School of Psychology and Clinical Language Sciences, University of Reading, UK

Daneman, D., Jones, J., Colton, P., Rydall, A., Maharaj, S., & Olmsted, M. (2002). Eating Disorders in Adolescent Girls and Young Adult Women with Type 1 Diabetes. *Diabetes Spectrum*, *15*(2), 83-105.

Daneman, D., Olmsted, M., Rydall, A., Maharaj, S., & Rodin, G. (1998). Eating disorders in young women with type 1 diabetes - Prevalence, problems and prevention. *HORMONE RESEARCH*, *50*, 79-86. https://doi.org/doi:10.1159/000053110

Darbar, N., & Mokha, M. (2008). Diabulimia: A body-image disorder in patients with type 1 diabetes mellitus. *Athletic Therapy Today*, *13*(4), 31-33. https://doi.org/doi:10.1123/att.13.4.31

Davidson, J. (2014). Diabulimia: how eating disorders can affect adolescents with diabetes. *Nursing standard (Royal College of Nursing (Great Britain) : 1987)*, *29*(2), 44-49. https://doi.org/doi:10.7748/ns.29.2.44.e7877

*De Paoli, T., & Rogers, P. J. (2018). Disordered eating and insulin restriction in type 1 diabetes: A systematic review and testable model. *Eating Disorders*, *26*(4), 343-360. https://doi.org/doi:10.1080/10640266.2017.1405651

*Dean, Y. E., Motawea, K. R., Aslam, M., Loayza Pintado, J. J., Popoola-Samuel, H. A. O., Salam, M., Prashant Obed Reddy, D., Webster, D., Aledani, E. M., Alqiqie, Z., Sultana, N., Alaa Ramadan Hussein, M., Elalem, A., Sidra Tahreem Hashmi, S., Mai Saad, M., Assal, M. W., Attia, N. M., Hagar, H., Heba Ahmed, A., . . . Aiash, H. (2024). Association Between Type 1 Diabetes Mellitus and Eating Disorders: A Systematic Review and Meta-Analysis. Endocrinology, Diabetes & Metabolism, 7(3). https://doi.org/doi:https://doi.org/10.1002/edm2.473

D'Emden, H., Holden, L., McDermott, B., Harris, M., Gibbons, K., Gledhill, A., & Cotterill, A. (2013). Disturbed eating behaviours and thoughts in Australian adolescents with type 1 diabetes. *Journal of Paediatrics and Child Health*, *49*(4), E317-E323. https://doi.org/doi:10.1111/jpc.12014

D'Emden, H., McDermott, B., Gibbons, K., Harris, M., & Cotterill, A. (2015). Choosing a screening tool to assess disordered eating in adolescents with type 1 diabetes mellitus. *Journal of Diabetes and its Complications*, *29*(1), 2-4. https://doi.org/doi:10.1016/j.jdiacomp.2014.09.008

Doyle, E. A. (2016). Screening for disordered eating behaviors in adolescents and young adults with type 1 diabetes. *Pediatric Nursing*, *42*(4), 197-200.

*Dunning, P. L. (1995). Young-Adult Perspectives of Insulin-Dependent Diabetes. *The Diabetes Educator*, *21*(1), 58-65. https://doi.org/doi:10.1177/014572179502100110

*Eilander, M. M., de Wit, M., Rotteveel, J., Aanstoot, H. J., Bakker-van Waarde, W. M., Houdijk, E. C., Nuboer, R., Winterdijk, P., & Snoek, F. J. (2017). Disturbed eating behaviors in adolescents with type 1 diabetes. How to screen for yellow flags in clinical practice? *Pediatr Diabetes*, *18*(5), 376-383. https://doi.org/10.1111/pedi.12400

Embaye, J., Hennekes, M., Snoek, F., & de Wit, M. (2024). Psychometric properties of the Diabetes Eating Problem Survey–Revised among Dutch adults with type 1 diabetes and implications for clinical use. Diabetic Medicine, 41(5). https://doi.org/doi:10.1111/dme.15313

*Embick, R. (2024). Turning the T1DE: Recovery factors in type 1 disordered eating. Dissertation Abstracts International: Section B: The Sciences and Engineering, 85(11), No Pagination Specified.

* akshelford@gmail.com

School of Psychology and Clinical Language Sciences, University of Reading, UK

Fairburn, C. G., Peveler, R., Davies, B., Mann, J. I., & Mayou, R. A. (1991). Eating disordered in young adults with insulin dependent diabetes mellitus: a controlled study. *British Medical Journal*, *303*, 17-20.

*Falcão, M. A., & Francisco, R. (2017). Diabetes, eating disorders and body image in young adults: an exploratory study about “diabulimia”. *Eating and Weight Disorders*, *22*(4), 675-682. https://doi.org/doi:https://doi.org/10.1007/s40519-017-0406-9

Fanik, R. (2014). Insulin manipulation and eating disorders in Young people with type 1 diabetes: implications for schools. *Journal of Diabetes Nursing*, *18*(6), 238-242.

Feinstein, A. (2008). *Body relationship as a predictor of self-care, physical health, and psychological well-being in adolescents with type 1 diabetes* (Publication Number 3377440) Alliant International University]. PROQUEST.

Ferrero Franco, R., Garcia de Lorenzo, A., & Gonzalez Castro, A. (2021). Diabulimia: An updated perspective. *Enferm Clin (Engl Ed)*, *31*(6), 396-397. https://doi.org/doi:10.1016/j.enfcle.2021.02.003

*Ferrey, A., Ashworth, G., Cabling, M., Rundblad, G., & Ismail, K. (2023). A thematic analysis of YouTube comments on a television documentary titled 'Diabulimia: The World's most dangerous eating disorder'. *Diabetic Medicine*, *40*(5), e15025. https://doi.org/doi:10.1111/dme.15025

Field-Lucas, E., & Carl Leith-van, H. (2019, 2019-11-27). P114 Recurrent a&e attendances? remember the psychosocial history. Archives of Disease in Childhood,

Figueiredo, J., Pavin, E. J., Argenton, J., Trevisan, T., & Silveira, M. S. V. (2023, 2023-2). DISORDERED EATING BEHAVIORS IN BRAZILIAN PERSONS WITH TYPE 1 DIABETES: FREQUENCY, INSULIN OMISSION, DEPRESSIVE SYMPTOMS, BODY SHAPE CONCERNS, AND CLINICAL OUTCOMES. DIABETES TECHNOLOGY & THERAPEUTICS,

Franke, P. (2014). Eating disorders and insulin misuse in people with type 1 diabetes. *Journal of Diabetes Nursing*, *18*(3), 92-98.

Franko, D., Weinger, K., Goebel-Fabbri, A., Rodriguez, M., Fikkan, J., Pearson, K., & Anderson, B. (2003). Insulin omission in women with type 1 diabetes: Validity of the Eating Disorder Inventory.*DIABETES* 63rd Scientific Sessions of the American Diabetes Association,

Gawlik, N. R., Elias, A. J., & Bond, M. J. (2016). Appearance Investment, Quality of Life, and Metabolic Control Among Women with Type 1 Diabetes. *International Journal of Behavioral Medicine*, *23*(3), 348-354. https://doi.org/doi:https://doi.org/10.1007/s12529-015-9524-9

*Goddard, G., & Oxlad, M. (2022). Caring for individuals with Type 1 Diabetes Mellitus who restrict and omit insulin for weight control: Evidence-based guidance for healthcare professionals. *Diabetes Res Clin Pract*, *185*, 109783. https://doi.org/doi:10.1016/j.diabres.2022.109783

*Goddard, G., & Oxlad, M. (2023). Insulin restriction or omission in Type 1 Diabetes Mellitus: a meta-synthesis of individuals' experiences of diabulimia. *Health Psychol Rev*, *17*(2), 227-246. https://doi.org/doi:10.1080/17437199.2021.2025133

*Goddard, G., Oxlad, M., & Turnbull, D. (2023). The misuse of insulin by males with Type 1 Diabetes Mellitus for weight and/or shape control: a systematic scoping review. *Journal of Diabetes and Metabolic Disorders*, *22*(1), 13-34. https://doi.org/doi:10.1007/s40200-022-01151-8

* akshelford@gmail.com

School of Psychology and Clinical Language Sciences, University of Reading, UK

Goebel-Fabbri, A. E. (2008). Insulin Restriction and Associated Morbidity and Mortality in Women with Type 1 Diabetes. *Diabetes Care*, *31*(3), 415-419. https://doi.org/doi:10.2337/dc07-2026

Goebel-Fabbri, A. E. (2008). Insulin Restriction and Associated Morbidity and Mortality in Women with Type 1 Diabetes. *Diabetes Care*, *31*(3), 415-419. https://doi.org/doi:10.2337/dc07-2026

*Goebel-Fabbri, A. E., Anderson, B. J., Fikkan, J., Franko, D. L., Pearson, K., & Weinger, K. (2011). Improvement and emergence of insulin restriction in women with type 1 diabetes. *Diabetes Care*, *34*(3), 545-550. https://doi.org/doi:10.2337/dc10-1547

Goebel-Fabbri, A. E., Fikkan, J., Connell, A., Vangsness, L., & Anderson, B. J. (2002). Identification and treatment of eating disorders in women with type 1 diabetes mellitus. *Treatments in Endocrinology*, *1*(3), 155-162. https://doi.org/doi:10.2165/00024677-200201030-00003

Goebel-Fabbri, A., Copeland, P., Touyz, S., & Hay, P. (2019). EDITORIAL: Eating disorders in diabetes: Discussion on issues relevant to type 1 diabetes and an overview of the Journal's special issue. *Journal of Eating Disorders*, *7*, 27. https://doi.org/doi:https://doi.org/10.1186/s40337-019-0256-0

*Goebel-Fabbri, A., Uplinger, N., Gerken, S., Mangham, D., Criego, A., & Parkin, C. (2009). Outpatient Management of Eating Disorders in Type 1 Diabetes. *Diabetes Spectrum*, *22*(3), 147-152.

*Gottesman, K., Ziegler, J., & Parker, A. (2015). Insulin Omission for Weight Control in Adolescents With Type 1 Diabetes Mellitus. *Topics in Clinical Nutrition*, *30*(4), 314-323. https://doi.org/doi:10.1097/TIN.0000000000000050

*Haagen, B. F. (2011). Insulin omission: A troubling trend among adolescent girls. *Journal of Psychosocial Nursing and Mental Health Services*, *49*(2), 6-7.

Hacia, S., Cichoń, L., Nowak, M., Fuchs, A., Fuchs, P., & Janas-Kozik, M. (2013). Autoaggressive behaviour with patients suffering from type 1 diabetes treated at the Clinic for Psychiatry and Psychotherapy Developmental Age - Description of cases. *Psychiatria Polska*, *47*(5), 887-896.

Hall, R., Keeble, L., Sunram-Lea, S. I., & To, M. (2021). A review of risk factors associated with insulin omission for weight loss in type 1 diabetes. *Clin Child Psychol Psychiatry*, *26*(3), 606-616. https://doi.org/doi:10.1177/13591045211026142

Hanlan, M. E., Griffith, J., Patel, N., & Jaser, S. S. (2013). Eating Disorders and Disordered Eating in Type 1 Diabetes: Prevalence, Screening, and Treatment Options. *Current Diabetes Reports*, *13*(6), 909-916. https://doi.org/doi:https://doi.org/10.1007/s11892-013-0418-4

*Harrison, A., Zaremba, N., Brown, J., Allan, J., Konstantara, E., Hopkins, D., Treasure, J., Ismail, K., & Stadler, M. (2021). A cognitive behavioural model of the bidirectional relationship between disordered eating and diabetes self care in people with type 1 diabetes mellitus. *Diabet Med*, *38*(7), e14578. https://doi.org/10.1111/dme.14578

*Hastings, A., McNamara, N., Allan, J., & Marriott, M. (2016). The importance of social identities in the management of and recovery from 'Diabulimia': A qualitative exploration. *Addictive Behaviors Reports*, *4*, 78-86. https://doi.org/doi:10.1016/j.abrep.2016.10.003

*Herpertz, S., Albus, C., Kielmann, R., Hagemann-Patt, H., Lichtblau, K., Köhle, K., Mann, K., & Senf, W. (2001). Comorbidity of diabetes mellitus and eating disorders: A follow-up study. *Journal of Psychosomatic Research*, *51*(5), 673-678. https://doi.org/doi:10.1016/S0022-3999(01)00246-X

* akshelford@gmail.com

School of Psychology and Clinical Language Sciences, University of Reading, UK

Herpertz, S., Albus, C., Wagener, R., Kocnar, M., Wagner , R., Henning, A., Best, F., Foerster, H., Schulze Schleppinghoff, B., Thomas, W., Kohle, K., Mann, K., & Senf, W. (1998). Comorbidity of Diabetes and Eating Disorders. *Diabetes Care*, *21*(7), 1110-1116.

Hockey, S., Brown, L. J., & Lunt, H. (1993). Prevalence of insulin self manipulation in young women with insulin dependent diabetes. *N Z Med J*, *106*(967), 474-476.

Hoffman, R. P. M. D. (2001). Eating disorders in adolescents with type 1 diabetes A closer look at a complicated condition. *Postgraduate Medicine*, *109*(4), 67.

*Howe, C. J., Jawad, A. F., Kelly, S. D., & Lipman, T. H. (2008). Weight-related concerns and behaviors in children and adolescents with type 1 diabetes. *Journal of the American Psychiatric Nurses Association*, *13*(6), 376-385. https://doi.org/doi:10.1177/1078390307310154

Hummadi, A., Yafei, S., Badedi, M., Abutaleb, R., Darraj, H., Alhagawy, A. J., Khawaji, A., Solan, Y., Alzughbi, T., Hakami, M., Jaddoh, S., Daghriri, A., & Khardali, M. (2023). Validation of the Arabic Version of Diabetes Eating Problem Survey–Revised (DEPS-R) among Adolescents with Type 1 Diabetes. *Nutrients*, *15*(3). https://doi.org/doi:10.3390/nu15030561

Iceta, S., Sohier, L., Bégin, C., Brazeau, A. S., Rabasa-Lhoret, R., & Gagnon, C. (2022). Impact of glycemic variability on cognitive impairment, disordered eating behaviors and self-management skills in patients with type 1 diabetes: study protocol for a cross-sectional online study, the Sugar Swing study. *BMC Endocrine Disorders*, *22*(1), 283. https://doi.org/doi:10.1186/s12902-022-01191-4

Ip, E. J., Doroudgar, S., Salehi, A., Salehi, F., & Najmi, M. (2023). Diabulimia: A Risky Trend Among Adults with Type 1 Diabetes Mellitus. *Endocrine Practice*, *29*(11), 849-854. https://doi.org/doi:10.1016/j.eprac.2023.08.001

*Jaensch, L., Goddard, G., Oxlad, M., & Franke, E. (2023). Health Professionals' Experiences Supporting People With Type 1 Diabetes Mellitus Who Deliberately Restrict and/or Omit Insulin for Weight, Shape, and/or Appearance: A Meta-synthesis. *Canadian Journal of Diabetes*, *47*(6), 532-542. https://doi.org/doi:10.1016/j.jcjd.2023.03.003

*Jain, V., Satapathy, A. K., & Yadav, J. (2015). Surreptitious insulin overdosing in adolescents with type 1 diabetes. *Indian Pediatrics*, *52*(8), 701-703. https://doi.org/doi:10.1007/s13312-015-0701-3

Jaser, S. S., Yates, H., Dumser, S., & Whittemore, R. (2011). Risky Business: Risk Behaviors in Adolescents With Type 1 Diabetes. *The Diabetes Educator*, *37*(6), 756-764. https://doi.org/doi:10.1177/0145721711422610

Jones, J. M., Lawson, M. L., Daneman, D., Olmsted, M. P., & Rodin, G. (2000). Eating disorders in adolescent females with and without type 1 diabetes: cross sectional study. *BMJ : British Medical Journal*, *320*(7249), 1563. https://doi.org/doi:https://doi.org/10.1136/bmj.320.7249.1563

Juruc, A., Kubiak, M., & Wierusz-Wysocka, B. (2016). Psychological and medical problems in prevention and treatment of eating disorders among people with type 1 diabetes. *Clinical Diabetology*, *5*(1), 26-31. https://doi.org/doi:10.5603/DK.2016.0005

Karastogiannidou, C., Giannoulaki, P., Samaras, I., Kotzakioulafi, E., Didangelos, T., Ioana Corina, B., & Vassilopoulou, E. (2021). The Diabetes Eating Problem Survey-Revised (DEPS-R) in a Greek

Adult Population with Type 1 Diabetes Mellitus: Model Comparison Supporting a Single Factor Structure. *Nutrients*, *13*(7), 2375. https://doi.org/doi:https://doi.org/10.3390/nu13072375

Kaufman, F. R. (2006). Consequences of weight gain associated with insulin therapy in adolescents. *Endocrinologist*, *16*(3), 155-162. https://doi.org/doi:10.1097/01.ten.0000217883.47474.84

Kelly, S. D., Howe, C. J., Hendler, J. P., & Lipman, T. H. (2005). Disordered eating behaviors in youth with type 1 diabetes. *Diabetes Educator*, *31*(4), 572-583. https://doi.org/doi:10.1177/0145721705279049

*Kennon, F., & Robinson, G. (2024). Parenting a child with 'Diabulimia': A systemic interpretative phenomenological analysis. Journal of Family Therapy, 46(3), 245-263. https://doi.org/doi:10.1111/1467-6427.12460

Khan, Y., & Montgomery, A. M. J. (1996). Eating attitudes in young females with diabetes: Insulin omission identifies a vulnerable subgroup. *British Journal of Medical Psychology*, *69*(4), 343-353. https://doi.org/doi:10.1111/j.2044-8341.1996.tb01877.x

*Kinik, M. F., Gonullu, F. V., Vatansever, Z., & Karakaya, I. (2017). Diabulimia, a Type I diabetes mellitus-specific eating disorder. *Turk Pediatri Ars*, *52*(1), 46-49. https://doi.org/doi:10.5152/TurkPediatriArs.2017.2366

*Kirkpatrick-Justice, K. A. (2004). *Eating disorders in adolescents with type 1 and type 2 diabetes mellitus: Prevelance and adherence to the regimen* University of Florida]. https://www.proquest.com/docview/305325209?pq-origsite=gscholar&fromopenview=true&sourcetype=Dissertations%20&%20Theses

Lamisse, F. (2006). Frequency and severity of eating disorders in young diabetics of type 1 diabetes mellitus: Review of literature. *Cahiers de Nutrition et de Dietetique*, *41*(1), 17-22. https://doi.org/doi:10.1016/s0007-9960(06)70603-4

Larrañaga, A., Docet, M. F., & García-Mayor, R. V. (2011). Disordered eating behaviors in type 1 diabetic patients. *World J Diabetes*, *2*(11), 189-195. https://doi.org/doi:10.4239/wjd.v2.i11.189

Lazo, C., & Agüero, S. D. (2019). The effect of diabetes mellitus diagnosis and its complication with eating disorders. *REVISTA CHILENA DE NUTRICION*, *46*(3), 352-360. https://doi.org/doi:10.4067/S0717-75182019000300352

Leal, L., da Silva, L., Moreira, L., Paiva, A., do Carmo Martins, M., Moreira-Araujo, R., & Frota, K. (2024). Prevalence of diabulimia and risk factors in people with type 1 diabetes: a systematic review.

Lee-Akers, D., Simon, J., & Akers, E. (2019, 2019-11-22). 887-P: Biological and Psychological Risk Factors for Eating Disorders in Type 1 Diabetes. Diabetes, suppl. Supplement 1,

Levek, N., Faruge-Hadiga, R., & Pinhas-Hamiel, O. (2023). Insulin Omission for Weight Loss in a Female Adolescent Treated With Advanced Hybrid Closed-Loop System: A Word of Caution. *Diabetes Care*, *46*(8), e143-e145. https://doi.org/doi:10.2337/dc23-0204

Lok, C. W., Wong, M. C., Yip, K. W., Ching, W. K., & Choi, E. K. Y. (2023). Validation of the traditional Chinese version of the diabetes eating problem survey-revised and study of the prevalence of disordered eating patterns in Chinese patients with type 1 DM. *BMC Psychiatry*, *23*(1), 382. https://doi.org/doi:10.1186/s12888-023-04744-6

* akshelford@gmail.com

School of Psychology and Clinical Language Sciences, University of Reading, UK

*Lorettu, L., Pes, G. M., Dore, M. P., Milia, P., & Nivoli, A. (2020). Eating disorders and diabetes: Behavioural patterns and psychopathology. Two case reports. *Rivista di Psichiatria*, *55*(4), 240-244. https://doi.org/doi:10.1708/3417.34001

*Luyckx, K., Verschueren, M., Palmeroni, N., Goethals, E. R., Weets, I., & Claes, L. (2019). Disturbed Eating Behaviors in Adolescents and Emerging Adults With Type 1 Diabetes: A One-Year Prospective Study. *Diabetes Care*, *42*(9), 1637-1644. https://doi.org/doi:10.2337/dc19-0445

Lv, W., Zhong, Q., Guo, J., Luo, J., Dixon, J., & Whittemore, R. (2021). Instrument context relevance evaluation, translation, and psychometric testing of the diabetes eating problem survey-revised (DEPS-R) among people with type 1 diabetes in China. *International Journal of Environmental Research and Public Health*, *18*(7). https://doi.org/doi:10.3390/ijerph18073450

*Major, B. D., Partridge, H. D., Zaidman, S. D., Eveleigh Nicholson, D., & Figueiredo, C. D. (2024, 2024-8-1). ComPASSION: A Screening Tool for Type 1 Diabetes and Disordered Eating (T1DE). BJPsych Open,

*Markowitz, J. T. P. H. D., Butler, D. A. M. S. W., Volkening, L. K. M. A., Antisdel, J. E. P. H. D., Anderson, B. J. P. H. D., & Laffel, L. M. B. M. D. (2010). Brief Screening Tool for Disordered Eating in Diabetes: Internal consistency and external validity in a contemporary sample of pediatric patients with type 1 diabetes. *Diabetes Care*, *33*(3), 495-500. https://doi.org/doi:https://doi.org/10.2337/dc09-1890

*Markowitz, J. T., Alleyn, C. A., Phillips, R., Muir, A., Young-Hyman, D., & Laffel, L. M. (2013). Disordered eating behaviors in youth with type 1 diabetes: prospective pilot assessment following initiation of insulin pump therapy. *Diabetes Technol Ther*, *15*(5), 428-433. https://doi.org/doi:10.1089/dia.2013.0008

*Martin, R., Davis, A., Pigott, A., & Cremona, A. (2023). A scoping review exploring the role of the dietitian in the identification and management of eating disorders and disordered eating in adolescents and adults with type 1 diabetes mellitus. *Clinical Nutrition ESPEN*, *58*, 375-387.

*Matthews, V. (2019). *An exploration of the relationship between insulin misuse and eating disorder psychopathology in adults with type 1 diabetes* (Publication Number 27774817) University of East Anglia (United Kingdom)]. England. https://www.proquest.com/dissertations-theses/exploration-relationship-between-insulin-misuse/docview/2333876074/se-2?accountid=13460 https://ueaeprints.uea.ac.uk/72707/

McCarvill, R., & Weaver, K. (2014). Primary care of female adolescents with type 1 diabetes mellitus and disordered eating. *Journal of Advanced Nursing*, *70*(9), 2005-2018. https://doi.org/doi:https://doi.org/10.1111/jan.12384

McConnell, E. M., Harper, R., Campbell, M., & Nelson, J. K. (2001). Achieving optimal diabetic control in adolescence: the continuing enigma. *DIABETES-METABOLISM RESEARCH AND REVIEWS*, *17*(1), 67-74. https://doi.org/doi:10.1002/1520-7560(200101/02)17:1<67::AID-DMRR175>3.0.CO;2-V

Mellin, A. E., Neumark-Sztainer, D., Patterson, J., & Sockalosky, J. (2004). Unhealthy weight management behavior among adolescent girls with type 1 diabetes mellitus: The role of familial eating patterns and weight-related concerns. *Journal of Adolescent Health*, *35*(4), 278-289. https://doi.org/doi:10.1016/S1054-139X(03)00535-4

* akshelford@gmail.com

School of Psychology and Clinical Language Sciences, University of Reading, UK

*Meltzer, L. J., Johnson, S. B., Prine, J. M., Banks, R. A., Desbrosiers, P. M., & Silverstein, J. H. (2001). Disordered Eating, Body Mass, and Glycaemic Control in Adolescents with Type 1 Diabetes. *Diabetes Care*, *24*, 678-682.

Merwin, R. M., Dmitrieva, N. O., Honeycutt, L. K., Moskovich, A. A., Lane, J. D., Zucker, N. L., Surwit, R. S., Feinglos, M., & Kuo, J. (2015). Momentary Predictors of Insulin Restriction Among Adults With Type 1 Diabetes and Eating Disorder Symptomatology. *Diabetes Care*, *38*(11), 2025. https://doi.org/doi:https://doi.org/10.2337/dc15-0753

*Merwin, R. M., Dmitrieva, N. O., Moskovich, A. A., Warnick, J. L., Ann, E. G. F., Lisa Swartz, T., & Darling, K. E. (2024). Profiles of disordered eating behaviour in type 1 diabetes using the DEPS-R and behaviour and glycaemic outcomes in a real-life setting. Diabetic Medicine, 41(6). https://doi.org/https://doi.org/10.1111/dme.15314

Merwin, R. M., Moskovich, A. A., Dmitrieva, N. O., Pieper, C. F., Honeycutt, L. K., Zucker, N. L., Surwit, R. S., & Buhi, L. (2014). Disinhibited eating and weight-related insulin mismanagement among individuals with type 1 diabetes. *Appetite*, *81*, 123-130. https://doi.org/doi:10.1016/j.appet.2014.05.028

*Merwin, R. M., Moskovich, A. A., Honeycutt, L. K., Lane, J. D., Feinglos, M., Surwit, R. S., Zucker, N. L., Dmitrieva, N. O., Babyak, M. A., Batchelder, H., & Mooney, J. (2018). Time of Day When Type 1 Diabetes Patients With Eating Disorder Symptoms Most Commonly Restrict Insulin. *Psychosomatic Medicine*, *80*(2), 222-229. https://doi.org/doi:10.1097/PSY.0000000000000550

*Moosavi, M., Kreisman, S., & Hall, L. (2015). Intentional Hypoglycemia to Control Bingeing in a Patient with Type 1 Diabetes and Bulimia Nervosa. *Canadian Journal of Diabetes*, *39*(1), 16-17. https://doi.org/doi:10.1016/j.jcjd.2014.04.007

Natarajan, N., & Obousy, S. (2024, 2024-8-1). Is Body Dissatisfaction a Risk Factor for Diabulimia and How Is It Assessed? A Rapid Systematic Review. BJPsych Open,

Neumark-Sztainer, D., Patterson, J., Mellin, A., Ackard, D. M., Utter, J., Story, M., & Sockalosky, J. (2002). Weight control practices and disordered eating behaviors among adolescent females and males with type 1 diabetes - Associations with sociodemographics, weight concerns, familial factors, and metabolic outcomes. *Diabetes Care*, *25*(8), 1289-1296. https://doi.org/doi:10.2337/diacare.25.8.1289

*Nielsen, S. (2002). Eating disorders in females with type 1 diabetes: An update of a meta-analysis. *European Eating Disorders Review*, *10*(4), 241-254. https://doi.org/doi:10.1002/erv.474

*Niemelä, P. E., Leppänen, H. A., Voutilainen, A., Möykkynen, E. M., Virtanen, K. A., Ruusunen, A. A., & Rintamäki, R. M. (2024). Prevalence of eating disorder symptoms in people with insulin-dependent-diabetes: A systematic review and meta-analysis. Eat Behav, 53, 101863. https://doi.org/doi:10.1016/j.eatbeh.2024.101863

*Nip, A. S. Y., Reboussin, B. A., Dabelea, D., Bellatorre, A., Mayer-Davis, E. J., Kahkoska, A. R., Lawrence, J. M., Peterson, C. M., Dolan, L., & Pihoker, C. (2019). Disordered Eating Behaviors in Youth and Young Adults With Type 1 or Type 2 Diabetes Receiving Insulin Therapy: The SEARCH for Diabetes in Youth Study. *Diabetes Care*, *42*(5), 859. https://doi.org/doi:https://doi.org/10.2337/dc18-2420

*Olmsted, M. P., Daneman, D., Rydall, A. C., Lawson, M. L., & Rodin, G. (2002). The effects of psychoeducation on disturbed eating attitudes and behavior in young women with type 1

* akshelford@gmail.com

School of Psychology and Clinical Language Sciences, University of Reading, UK

diabetes mellitus. *International Journal of Eating Disorders*, *32*(2), 230-239. https://doi.org/doi:10.1002/eat.10068

Osipoff, J. N., Sattar, N., Garcia, M., & Wilson, T. A. (2010). Prime-Time Hypoglycemia: Factitious Hypoglycemia During Insulin-Pump Therapy. *Pediatrics*, *125*(5), E1246-E1248. https://doi.org/doi:10.1542/peds.2009-1830

*Papadakis, J. L., Anderson, L. M., Vesco, A. T., Evans, M. A., & Weissberg-Benchell, J. (2019, 2019-6-1). Intentional Insulin Omission for Weight Loss and Psychosocial Outcomes among Youth with Type 1 Diabetes: Findings from Routine Screening. Diabetes,

Papelbaum, M., Appolinario, J. C., de Oliveira Moreira, R., Duchesne, M., Kupfer, R., & Coutinho, W. (2007). Distribution of eating disorders in subjects with type 1 and type 2 diabetes mellitus: A description of two cases. *29*(1), 93-96. https://doi.org/10.1590/S0101-81082007000100016

Partridge, H., Figueiredo, C., Rouse, L., Cross, C., Pinder, C., Ryder, J., Bennett, M., & Stacey, N. (2020). Type 1 diabetes and disordered eating (T1DE): the ComPASSION Project – Wessex. *Practical Diabetes*, *37*(4), 127-132. https://doi.org/doi:10.1002/pdi.2286

*Peducci, E., Mastrorilli, C., Falcone, S., Santoro, A., Fanelli, U., Iovane, B., Incerti, T., Scarabello, C., Fainardi, V., Caffarelli, C., Di Mauro, D., Dodi, I., Tchana, B., & Vanelli, M. (2019). Disturbed eating behavior in pre-teen and teenage girls and boys with type 1 diabetes. *Acta Biomed*, *89*(4), 490-497. https://doi.org/doi:10.23750/abm.v89i4.7738

Pereira, R. F., & Alvarenga, M. (2007). Disordered eating: Identifying, treating, preventing, and differentiating it from eating disorders. *Diabetes Spectrum*, *20*(3), 141-144+146. https://doi.org/doi:10.2337/diaspect.20.3.141

*Peterson, C. M., Fischer, S., & Young-Hyman, D. (2015). Topical review: a comprehensive risk model for disordered eating in youth with type 1 diabetes. *J Pediatr Psychol*, *40*(4), 385-390. https://doi.org/doi:10.1093/jpepsy/jsu106

Peveler, R. C., Fairburn, C. G., Boller, I., & Dunger, D. B. (1992). Eating Disorders in Adolescents with IDDM: A controlled study. *Diabetes Care*, *15*(10), 1356-1360. https://doi.org/doi:10.2337/diacare.15.10.1356

Philippi, S. T., Cardoso, M. G. L., Koritar, P., & Alvarenga, M. (2013). Risk behaviors for eating disorder in adolescents and adults with type 1 diabetes. *Revista Brasileira de Psiquiatria*, *35*(2), 150-156. https://doi.org/doi:10.1590/1516-4446-2012-0780

Pinar, R. (2005). Disordered eating behaviors among Turkish adolescents with and without type 1 diabetes. *Journal of Pediatric Nursing*, *20*(5), 383-388. https://doi.org/doi:10.1016/j.pedn.2005.07.001

*Pinhas-Hamiel, O. M. D., Hamiel, U. M. D., Greenfield, Y. B., Boyko, V. M., Graph-Barel, C. M. D., Rachmiel, M. M. D., Lerner-Geva, L. M. D., & Reichman, B. M. (2013). Detecting intentional insulin omission for weight loss in girls with type 1 diabetes mellitus. *International Journal of Eating Disorders*, *46*(8), 819. https://doi.org/doi:10.1002/eat.22138

Pinhas-Hamiel, O., & Levy-Shraga, Y. (2013). Eating Disorders in Adolescents with Type 2 and Type 1 Diabetes. *Current Diabetes Reports*, *13*(2), 289-297. https://doi.org/doi:10.1007/s11892-012-0355-7

* akshelford@gmail.com

School of Psychology and Clinical Language Sciences, University of Reading, UK

Pinhas-Hamiel, O., Hamiel, U., & Levy-Shraga, Y. (2015). Eating disorders in adolescents with type 1 diabetes: Challenges in diagnosis and treatment. *WORLD JOURNAL OF DIABETES*, *6*(3), 517-526. https://doi.org/doi:10.4239/wjd.v6.i3.517

Polonsky, W. H., Anderson, B. J., Lohrer, P. A., Aponte, J. E., Jacobson, A. M., & Cole, C. F. (1994). Insulin omission in women with IDDM. *Diabetes Care*, *17*(10), 1178-1185. https://doi.org/doi:10.2337/diacare.17.10.1178

*Poos, S., Faerovitch, M., Pinto, C., Jamalkhani, N., Chaudhri, F., Khan, S., Lo, D. F., McGowan, K., & Martin, A. (2023). The role of diabetes distress in Diabulimia. *J Eat Disord*, *11*(1), 213. https://doi.org/doi:10.1186/s40337-023-00924-7

Prasad-Reddy, L. (2012). Disordered eating in type 1 diabetes: Insulin omission and diabulimia. *U.S. Pharmacist*, *37*(5).

*Priesterroth, L., Grammes, J., Clauter, M., & Kubiak, T. (2021). Diabetes technologies in people with type 1 diabetes mellitus and disordered eating: A systematic review on continuous subcutaneous insulin infusion, continuous glucose monitoring and automated insulin delivery. *Diabetic Medicine*, *38*(7), e14581. https://doi.org/doi:10.1111/dme.14581

Priesterroth, L., Grammes, J., Strohm, E. A., & Kubiak, T. (2022). Disordered eating behaviours and eating disorders in adults with type 1 diabetes (DEBBI): rational and design of an observational longitudinal online study [Article]. *BMJ Open*, *12*(9), e064863, Article e064863. https://doi.org/10.1136/bmjopen-2022-064863

*Racicka, E., & Bryńska, A. (2015). Eating disorders in children and adolescents with type 1 and type 2 diabetes - prevalence, risk factors, warning signs. *Psychiatria Polska*, *49*(5), 1017-1024. https://doi.org/doi:10.12740/PP/39536

*Rancourt, D., Foster, N., Bollepalli, S., Fitterman-Harris, H. F., Powers, M. A., Clements, M., & Smith, L. B. (2019). Test of the modified dual pathway model of eating disorders in individuals with type 1 diabetes. *International Journal of Eating Disorders*, *52*(6), 630-642. https://doi.org/doi:10.1002/eat.23054

Rodin, G., & Daneman, D. (1992). Eating Disorders and IDDM: A problematic association. *Diabetes Care*, *15*(10), 1402-1412. https://doi.org/doi:10.2337/diacare.15.10.1402

Rodin, G., Olmsted, M. P., Rydall, A. C., Maharaj, S. I., Colton, P. A., Jones, J. M., Biancucci, L. A., & Daneman, D. (2002). Eating disorders in young women with type 1 diabetes mellitus. *Journal of Psychosomatic Research*, *53*(4), 943-949. https://doi.org/doi:10.1016/S0022-3999(02)00305-7

Rodríguez, J. H., & Llanes, L. L. (2020). Clinical behavior and therapeutic approach of eating disorders in people with type 1 diabetes mellitus. *Revista Cubana de Medicina General Integral*, *36*(2), 1-15.

Roney, A. M., & DeBoor, S. (2015). *An Exploration of Eating Disorders (Diabulimia) Associated with Type 1 Diabetes* (Publication Number 1591403) University of Nevada, Reno]. United States -- Nevada.

*Ruelens, C., & Vrieze, E. (2022). Diabetes mellitus and eating disorders: modified treatment is necessary. *Tijdschrift voor Psychiatrie*, *64*(2022), 220-225.

Ruth-Sahd, L. A., Schneider, M., & Haagen, B. (2009). Diabulimia: What it is and how to recognise it in critical care. *Dimensions of Critical Care Nursing*, *28*(4), 147-153.

* akshelford@gmail.com

School of Psychology and Clinical Language Sciences, University of Reading, UK

*Rydall, A. C., Rodin, G. M., Olmsted, M. P., Devenyi, R. G., & Daneman, D. (1997). Disordered eating behavior and microvascular complications in young women with insulin-dependent diabetes mellitus. *New England Journal of Medicine*, *336*(26), 1849-1854. https://doi.org/doi:10.1056/NEJM199706263362601

Sassmann, H., Albrecht, C., Busse-Widmann, P., Hevelke, L. K., Kranz, J., Markowitz, J. T., Marshall, L. F., Meurs, S., Soye, I. H., & Lange, K. (2015). Psychometric properties of the German version of the Diabetes Eating Problem Survey-Revised: additional benefit of disease-specific screening in adolescents with Type 1 diabetes. *Diabetic Medicine*, *32*(12), 1641-1647. https://doi.org/doi:https://doi.org/10.1111/dme.12788

Schmitt, T. L. (2012). Disordered eating in adolescent females with T1DM. *Nurse Practitioner*, *37*(9), 38-42. https://doi.org/doi:10.1097/01.NPR.0000418382.98982.f5

*Schober, E., Wagner, G., Berger, G., Gerber, D., Mengl, M., Sonnenstatter, S., Barrientos, I., Rami, B., Karwautz, A., & Fritsch, M. (2011). Prevalence of intentional under- and overdosing of insulin in children and adolescents with type 1 diabetes. *Pediatric Diabetes*, *12*(7), 627-631. https://doi.org/doi:10.1111/j.1399-5448.2011.00759.x

*Schwartz, S. A. (2003). *Disordered eating in female adolescents with insulin dependent diabetes mellitus* (Publication Number 8)

Schwimmer, K. J., & Swope, A. (2014). *Insulin Omission in Adolescents with Type I Diabetes: A Model Group Intervention* (Publication Number 3630181) Alliant International University]. United States -- California.

*Shelford, A., Wood, L., & Tomlin, A. (2024). *Interventions for insulin omission for weight loss in people with type 1 diabetes without a clinical eating disorder: a systematic review.* PsyArXiv. https://doi.org/10.31234/osf.io/nkdc8

Simmons, A., McMahon, L., Crosbie, V., & Carlson, L. (2021). A multidisciplinary team approach to screening, assessment and early intervention for young people with type 1 diabetes and disordered eating behaviour. *Clinical Child Psychology and Psychiatry*, *26*(3), 629-642. https://doi.org/doi:10.1177/13591045211013872

*Snyder, L. L., Truong, Y. K.-N., & Law, J. R. (2016). Evaluating substance use and insulin misuse in adolescents with type 1 diabetes. *The Diabetes Educator*, *42*(5), 529-537. https://doi.org/https://dx.doi.org/10.1177/0145721716659149

*Stadler, M., Rosenthal, M., Turner, D., Brown, J., Harrison, A., Liu, Y. F., Oliver, N., Konstantara, E., Hopkins, D., Treasure, J., & Ismail, K. (2023, 2023-9). Outcomes of the multidisciplinary 'Severe disordered eating in type 1 diabetes (T1DE)' pan-London service. DIABETOLOGIA,

*Staite, E., Zaremba, N., Macdonald, P., Allan, J., Treasure, J., Ismail, K., & Stadler, M. (2018). 'Diabulima' through the lens of social media: a qualitative review and analysis of online blogs by people with Type 1 diabetes mellitus and eating disorders. *Diabetic Medicine*, *35*(10), 1329-1336. https://doi.org/doi:https://doi.org/10.1111/dme.13700

Stancin, T., Link, D., & Reuter, J. (1989). Binge Eating and Purging in Young Women With IDDM. *Diabetes Care*, *12*, 601-603.

* akshelford@gmail.com

School of Psychology and Clinical Language Sciences, University of Reading, UK

Starkey, K., & Wade, T. (2010). Disordered eating in girls with Type 1 diabetes: Examining directions for prevention. *Clinical Psychologist*, *14*(1), 2-9. https://doi.org/doi:https://doi.org/10.1080/13284201003660101

Streiegel-Moore, R. H., Nicholson, T. J., & Tamborlane, W. (1992). Prevelance of Eating Disorder Symptoms in Preadolescent and Adolescent Girls with IDDM. *Diabetes Care*, *15*(10), 1361-1368.

Szmukler, G. I. (1984). Anorexia Nervosa and Bulimia in Diabetics. *Journal of Psychosomatic Research*, *28*(1), 365-369.

*Szmukler, G. I., & Russell, G. F. (1983). Diabetes mellitus, anorexia nervosa and bulimia. *British Journal of Psychiatry*, *142*, 305-308. https://doi.org/10.1192/bjp.142.3.305

*Takii, M., Komaki, G., Uchigata, Y., Maeda, M., Omori, Y., & Kubo, C. (1999). Differences between bulimia nervosa and binge-eating disorder in females with type 1 diabetes: The important role of insulin omission. *Journal of Psychosomatic Research*, *47*(3), 221-231. https://doi.org/doi:10.1016/S0022-3999(99)00031-8

*Takii, M., Uchigata, Y., Nozaki, T., Nishikata, H., Kawai, K., Komaki, G., Iwamoto, Y., & Kubok, C. (2002). Classification of type 1 diabetic females with bulimia nervosa into subgroups according to purging behavior. *Diabetes Care*, *25*(9), 1571-1575. https://doi.org/doi:10.2337/diacare.25.9.1571

*Takii, M., Uchigata, Y., Tokunaga, S., Amemiya, N., Kinukawa, N., Nozaki, T., Iwamoto, Y., & Kubo, C. (2008). The duration of severe insulin omission is the factor most closely associated with the microvascular complications of type 1 diabetic females with clinical eating disorders. *International Journal of Eating Disorders*, *41*(3), 259-264. https://doi.org/doi:10.1002/eat.20498

Tierney, S., Deaton, C., & Whitehead, J. (2009). Caring for people with type 1 diabetes mellitus engaging in disturbed eating or weight control: a qualitative study of practitioners' attitudes and practices. *Journal of Clinical Nursing*, *18*(3), 384. https://doi.org/doi:10.1111/j.1365-2702.2008.02434.x

*Treasure, J., Kan, C., Stephenson, L., Warren, E., Smith, E., Heller, S., & Ismail, K. (2015). Developing a theoretical maintenance model for disordered eating in Type 1 diabetes. *Diabet Med*, *32*(12), 1541-1545. https://doi.org/10.1111/dme.12839

*Troncone, A., Affuso, G., Cascella, C., Chianese, A., Pizzini, B., Zanfardino, A., Iafusco, D., Lera, R., Bracciolini, G. P., Grosso, C., Bertelli, E., Cherubini, V., Piccinno, E., Delvecchio, M., Ortolani, F., Vendemiale, M., Rutigliano, A., Zecchino, C., Zucchini, S., . . . Longo, B. (2022). Prevalence of disordered eating behaviors in adolescents with type 1 diabetes: Results of multicenter Italian nationwide study. *International Journal of Eating Disorders*, *55*(8), 1108-1119. https://doi.org/doi:10.1002/eat.23764

*Troncone, A., Affuso, G., Cascella, C., Chianese, A., Zanfardino, A., Iafusco, D., Lera, R., Bracciolini, G. P., Grosso, C., Bertelli, E., Cherubini, V., Piccinno, E., Delvecchio, M., Ortolani, F., Vendemiale, M., Rutigliano, A., Zecchino, C., Zucchini, S., Maltoni, G., . . . Longo, B. (2023). Prevalence and Multidimensional Model of Disordered Eating in Youths With Type 1 Diabetes: Results From a Nationwide Population-Based Study. *Journal of Pediatric Psychology*, *48*(9), 731-739. https://doi.org/doi:10.1093/jpepsy/jsad016

* akshelford@gmail.com

School of Psychology and Clinical Language Sciences, University of Reading, UK

Troncone, A., Cascella, C., Chianese, A., Zanfardino, A., Piscopo, A., Borriello, A., Casaburo, F., del Giudice, E. M., & Iafusco, D. (2020). Body Image Problems and Disordered Eating Behaviors in Italian Adolescents With and Without Type 1 Diabetes: An Examination With a Gender-Specific Body Image Measure. *Frontiers in Psychology*, *11*, 556520. https://doi.org/doi:10.3389/fpsyg.2020.556520

*Troncone, A., Chianese, A., Zanfardino, A., Cascella, C., Confetto, S., Piscopo, A., Loffredo, G., Golino, A., & Iafusco, D. (2020). Disordered Eating Behaviors Among Italian Adolescents with Type 1 Diabetes: Exploring Relationships with Parents' Eating Disorder Symptoms, Externalizing and Internalizing Behaviors, and Body Image Problems. *Journal of Clinical Psychology in Medical Settings*, *27*(4), 727-745. https://doi.org/doi:10.1007/s10880-019-09665-9

Urbanski, P., Goebel-Fabbri, A., Powers, M. A., & Taylor, D. (2009). The Diabetes Educator's Role in Managing Eating Disorders and Diabetes. *Diabetes Spectrum*, *22*(3), 159-162.

van Heyningen, C. D. L., & Manoharan, K. S. (2018). Diabulimia: an easily missed diagnosis? *BRITISH JOURNAL OF DIABETES*, *18*(4), 167-170. https://doi.org/doi:10.15277/bjd.2018.196

*Wakelin, K. E., Read, R. K., O'Donnell, N., Baker, M., Satherley, R. M., Stewart, R., & Jones, C. J. (2023). Integrating conversations about disordered eating in children and young people into routine type 1 diabetes care: a practical guide. *Practical Diabetes*, *40*(4), 11-17. https://doi.org/doi:10.1002/pdi.2464

Warnick, J. P., Darling, K. P., Topor, L. S. M. D. M., & Jelalian, E. P. (2022, 2022-3-28). Development of a Weight Management Program for Teens With Type 1 Diabetes and Overweight/Obesity. Obesity, suppl. SUPPLEMENT 1,

*Watt, A., Ng, A. H., Sandison, A., Fourlanos, S., & Bramley, A. (2022). Prevalence of disordered eating in adults with type 1 diabetes in an Australian metropolitan hospital. *Health and Social Care in the Community*, *30*(4), e974-e980. https://doi.org/doi:10.1111/hsc.13500

Wetter, S. E., & Driscoll, K. A. (2023). Commentary: From Recommendations to Reality: Assessment and Treatment of Adolescents With Type 1 Diabetes and Disordered Eating Behaviors. *Journal of Pediatric Psychology*, *48*(9), 740-742. https://doi.org/doi:10.1093/jpepsy/jsad036

*Wilkinson, K. (2019). *Risk and maintenance factors for disordered eating in children and young people with type 1 diabetes: a systematic review* [Systematic Review Protocol].

Wilson, V. (2012). *Reflections on reducing insulin to lose weight: NT* (Vol. 108). Emap Limited.

*Winston, A. P. (2020). Eating Disorders and Diabetes. *Current Diabetes Reports*, *20*(8), 32. https://doi.org/doi:https://doi.org/10.1007/s11892-020-01320-0

Wisting, L., & Snoek, F. (2020). Terminology matters: 'diabulimia' is insufficient to describe eating disorders in individuals with Type 1 diabetes. *Diabetic Medicine*, *37*(6), 1075-1076. https://doi.org/doi:10.1111/dme.14108

Wisting, L., Bang, L., Skrivarhaug, T., Dahl-Jørgensen, K., & Rø, Ø. (2015). Adolescents with Type 1 Diabetes – The Impact of Gender, Age, and Health-Related Functioning on Eating Disorder Psychopathology. *PLoS ONE*, *10*(11), e0141386. https://doi.org/doi:https://doi.org/10.1371/journal.pone.0141386

*Wisting, L., Froisland, D. H., Skrivarhaug, T., Dahl-Jorgensen, K., & Ro, O. (2013). Disturbed eating behavior and omission of insulin in adolescents receiving intensified insulin treatment: a

nationwide population-based study. *Diabetes Care*, *36*(11), 3382-3387. https://doi.org/doi:10.2337/dc13-0431

Wisting, L., Reas, D. L., Bang, L., Skrivarhaug, T., Dahl-Jørgensen, K., & Rø, Ø. (2017). Eating patterns in adolescents with type 1 diabetes: Associations with metabolic control, insulin omission, and eating disorder pathology. *Appetite*, *114*, 226-231. https://doi.org/doi:10.1016/j.appet.2017.03.035

Wisting, L., Wonderlich, J., Skrivarhaug, T., Dahl-Jorgensen, K., & Ro, O. (2019). Psychometric properties and factor structure of the diabetes eating problem survey - revised (DEPS-R) among adult males and females with type 1 diabetes. *J Eat Disord*, *7*, 2. https://doi.org/doi:10.1186/s40337-018-0232-0

*Yafei, S., Hummadi, A., Badedi, M., Darraj, H., Khawaji, A., Alzughbi, T., Abutaleb, R., Alhagawy, A. J., Alnami, A., Kudam, B., Bahsan, F., Kariri, M., Adawi, M., Daghriri, M., Hassan, R., Soeid, M., & Alzughbi, N. (2023). Disordered Eating Behaviors and Insulin Restriction in Saudi Adolescents and Young Adults with Type 1 Diabetes. *Medicina*, *59*(2), 345. https://doi.org/doi:https://doi.org/10.3390/medicina59020345

*Yahya, A. S., Khawaja, S., Chukwuma, J., & Chukwuma, C. (2020). Early Diagnosis and Management of Bulimia Nervosa in Type 1 Diabetes. *Primary Care Companion for CNS Disorders*, *22*(6). https://doi.org/doi:10.4088/PCC.20nr02707

Young-Hyman, D. L., & Davis, C. L. (2010). Disordered eating behavior in individuals with diabetes: importance of context, evaluation, and classification. *Diabetes Care*, *33*(3), 683-689. https://doi.org/doi:10.2337/dc08-1077

Zaldivar, R. A., Leavitt, J. A., Griepentrog, G. J., Woog, J. J., & Bradley, E. A. (2009). Rhino-orbital-cerebral mucormycosis: A lethal complication of body dysmorphic disorder [Article]. *Ophthalmic Plastic and Reconstructive Surgery*, *25*(5), 398-399. https://doi.org/10.1097/IOP.0b013e3181b54af3

*Zaremba, N., Harrison, A., Brown, J., Allan, J., Pillay, D., Treasure, J., Ayis, S., Hopkins, D., Ismail, K., & Stadler, M. (2024). Protocol for the STEADY intervention for type 1 diabetes and disordered eating: safe management of people with Type 1 diabetes and EAting Disorders studY. Diabetic Medicine. https://doi.org/doi:10.1111/dme.15273

Zaremba, N., Pillay, D., Harrison, A., Brown, J., Treasure, J., Hopkins, D., Ismail, K., & Stadler, M. (2023). Distribution of psychiatric disorders and subtypes of type 1 diabetes and disordered eating in the safe management of people with type 1 diabetes and eating disorders study (STEADY) feasibility randomised controlled trial. Diabetic Medicine

*Zaremba, N., Watson, A., Kan, C., Broadley, M., Partridge, H., Figuereido, C., Hopkins, D., Treasure, J., Ismail, K., Harrison, A., & Stadler, M. (2020). Multidisciplinary healthcare teams' challenges and strategies in supporting people with type 1 diabetes to recover from disordered eating. *Diabetic Medicine*, *37*(12), 1992-2000. https://doi.org/doi:10.1111/dme.14207

* akshelford@gmail.com

School of Psychology and Clinical Language Sciences, University of Reading, UK
