# Supplementary material for: Insulin-Related Disordered Eating Behaviour: A Scoping Review of Evidence
Source: Curr Diab Rep. 2026 Jul 29;26(1):23. doi: 10.1007/s11892-026-01637-2 (PMC13415336; doi:10.1007/s11892-026-01637-2)
Supplement: Supplementary file 5 [file 11892_2026_1637_MOESM5_ESM.pdf]

S5: Prevalence and measure details table

Table S5. Table of sources used for synthesis of results on prevalence of insulin-related disordered eating behaviour

Behaviour	Citation	n (Female)	Sample description	Point of prevalence	Prevalence [confidence intervals]	Measure (type)
**Restriction and inflation**						
	Dunning et al. 1995	n=59 (F=26)	Outpatient diabetes clinic in inner-city teaching hospital, PWT1D 17-30yo. Melbourne, Australia	Lifetime	41.0% restrict, 18.0% inflate	Custom (survey)
	Matthews et al. 2019	n=224 (F=171)	Adults with T1D>1y living in the UK recruited online through twitter and diabetes organisations	Current (past 7 d)	24.5% F, 6.4% M restrict<br>10.5% F, 6.4% M inflate	Custom (survey)
	Schober et al. 2011	n=241 (F=138)	PWT1D>1y, 10-22yo under care of 21 paediatric diabetes centres across Austria	Current	3.7% restrict<br>11.3% inflate	DSMP (interview)
	Snyder et al. 2016	n=60 (F=29)	PWT1D >1y, 12-20yo recruited from routine clinic appointments at University of North Carolina paediatric diabetes clinic, USA	Current	3.4% restrict<br>13.8% inflate	Custom (survey)
**Restriction**						
	Ackard et al. 2008	n=143 (F=70)	Youth with type 1 diabetes across Minnesota – data from AHEAD and Project EAT studies	Current	10.3% F, 1.4% M partial<br>7.4% F, 1.4% M total	AHEAD adaptation (survey)
	Albaladejo et al. 2023	n=198 (F=62)	T1D adults in France receiving at-home diabetes care managed with an insulin pump	Current	39.0% M [95%CI 30.8, 47.7]<br>37.7% F [95%CI 25.9, 51.1]	mSCOFF (survey)
	Baechle et al. 2019	n=1318 (F=734)	Germany-wide postal questionnaire, 6y longitudinal study. T1D onset <5yo with diabetes duration >10y, returning 2 consecutive questionnaires	Current	8.6% overall; 9.0% F, 8.2% M	Custom (survey)
	Battaglia et al. 2006	n=69	Young adult females with T1D attending diabetes clinic in USA	Current	10.1% overall	Others' custom (survey)

* akshelford@gmail.com

School of Psychology and Clinical Language Sciences, University of Reading, UK

Behaviour	Citation	n (Female)	Sample description	Point of prevalence	Prevalence [confidence intervals]	Measure (type)
	Beam et al. 2021	n=247	Highschool seniors with T1D in USA; secondary analysis of multisite dataset	Current	16.5% overall	Custom (survey)
	Biggs et al. 1994	n=42	Female adults with T1D recruited through private diabetes clinics, American Diabetes Association meetings/newsletters/adverts	Current	35.7% overall	Custom (interview)
	Bryden et al. 1999	n=65 (F=26)	Adolescents with T1D attending Oxford UK based diabetes clinics	Lifetime	30.0% F, 0.0% M	Custom (interview)
	Cantwell & Steel 1996	n=48	17-30yo females attending Royal Infirmary of Edinburgh for T1D	Unknown	20.8% overall	Custom (interview)
	Chou et al. 2023	n=110 (F=60)	Aged 10-30y diagnosed with T1D before 18yo, regularly tracked in a paediatric diabetes clinic in southern Taiwan	Current	11.0% overall	mSCOFF (survey)
	Coleman 2020	n=45 (F=42)	PWT1D and a history of insulin restriction for weight loss recruited through university campus adverts (Lancashire) and social media	Lifetime	38% overall	Others' custom (survey)
	Colton et al. 2015	n=71/126	Females with T1D receiving comprehensive care at the Hospital for Sick Children in Toronto, aged 9-13 at recruitment (<2004)	Current and Lifetime	26.8% overall<br>66% experience onset over 10-14 years	cEDE/EDE (interview)
	d'Emden et al. 2013	n=124 (F=66)	13-18yo with T1D attending routine appointment at diabetes clinic with a caregiver in Queensland, Australia	Current	7.6% F, 3.4% M	EDE-Q and EDI-3-RC adaptation (survey)
	Eilander et al. 2017	n=103 (F=53)	T1D youth aged 8-15 across 5 dutch diabetes centres	Current	15.5%	MY-Q; DEPS-R; AHEAD adaptation (survey)
	Fairburn et al. 1991	n=100 (F=54)	PWT1D aged 17-25yo on clinic register in Oxford, living in Oxfordshire	Lifetime	40.0% F	EDE adaptation (survey)

* akshelford@gmail.com

School of Psychology and Clinical Language Sciences, University of Reading, UK

Behaviour	Citation	n (Female)	Sample description	Point of prevalence	Prevalence [confidence intervals]	Measure (type)
	Falcao et al. 2017	n=55 (F=37)	Young adults with T1D recruited online, fluent in Portuguese, 18-30yo	Current	1.5% overall	Custom (survey)
	Gawlik et al. 2016	n=176	Females with T1D >6m aiming for mean age of 36yo recruited through online Australian diabetes support groups and local (Adelaide, Australian) education seminars	Lifetime	21.0%	Custom (survey)
	Goebel-Fabbri et al. 2011	n=207/390	Females with T1D >1y, 13-60yo recruited from routine diabetes clinic appointments	Current	35.7% overall	Custom (survey)
	Herpertz et al. 1998	n=341 (F=188)	18-65yo PWT1D across 12 diabetes centres in Essen and Cologne (Germany)	Unknown	5.9% overall	FSE adaptation (survey)
	Howe et al. 2008	n=295 (F=137)	Children over 11yo with T1D>1y under care at the Diabetes Centre in the Children's Hospital of Philadelphia	Lifetime	2.2% F, 0.0% M partial<br>1.5% F, 1.3% M total	Project EAT items (survey)
	Jones et al. 2000	n=361	Females aged 12-19y with T1D>1y identified from diabetes clinic lists across 3 children's hospitals approached over telephone, post, or clinic appointment	Current	11% overall	EDE adaptation (interview)
	Kirkpatrick-Justice, 2004	n=168 (F=71)	Adolescents age 10-21y with T1D recruited from paediatric endocrinology clinics in Florida and Missouri	Current	11.8% F overall, 0.0% M overall	Custom (difference score)
	Luyckz et al. 2019	n=300 (F=170)	Secondary analysis of data from Belgian Diabetes Registry: Dutch-speaking youth 16-28yo with T1D	Current	10.3% partial, 3.7% total	DEPS-R (survey)
	Markowitz et al. 2013	n=37 (F=19)	Youth aged 10-17y with T1D planning to initiate CSII from MDI management, recruited from 3 diabetes clinics across Massachusetts and Georgia, USA	Current	11% partial, 3% total overall	DEPS-R (survey)
	Mellin et al. 2004	n=30	Adolescent girls 13-20yo with T1D>1y followed by a paediatric diabetes clinic in Minnesota	Unknown	33.0% F partial, 46.0% F total	AHEAD adaptation (survey)

* akshelford@gmail.com

School of Psychology and Clinical Language Sciences, University of Reading, UK

Behaviour	Citation	n (Female)	Sample description	Point of prevalence	Prevalence [confidence intervals]	Measure (type)
	Meltzer et al. 2001	n=100 (F=46)	Adolescents 11-19yo with T1D>1y recruited from 2 diabetes clinics in Florida	Current	7.1% F, 4.9% M overall	EDI adaptation (survey)
	Merwin et al. 2018	n=59 (F=55)	Subset of 83 18-65yo PWT1D recruited from patient registries and flyers in diabetes clinics in North Carolina for 2015 study	Current (past 72 h)	56.9% overall "at least once"	Custom (verbal EMA)
	Nip et al. 2019	n=2,156 (F=1,528)	Analysis of data collected for SEARCH Cohort Study (phase 3) participants with T1D diagnosed at 10yo+ and completed DEPS-R	Current	18.2% overall	DEPS-R (survey)
	Peducci et al. 2019	n=85 (F=51)	Children with T1D >1y, 8-14yo from regional diabetes centre in Parma, Italy, all managing with MDI and a balanced meal plan	Current	21.5% F, 5.8% M	EDE adaptation (interview)
	Peveler et al. 1992	n=76 (F=33)	Adolescents 11-18yo with T1D on case register for diabetes outpatient clinic in Oxford	Lifetime	15.0% F	EDE adaptation (interview)
	Philippi et al. 2013	n=189 (F=141)	PWT1D>1y age 12-59yo under the care of 3 specialist diabetes clinics in Sao Paulo and Santos, Brazil	Unknown	11.3% F	Custom (survey)
	Pinar 2005	n=45 (F=24)	Adolescents 12-18yo with T1D>1y recruited from outpatient diabetes clinic in Istanbul.	Unknown	40.0% overall	Custom (survey)
	Polonsky et al. 1994	n=341	Females with T1D>1y aged 13-60yo from diabetes centre in Massachusetts	Current	12.6% overall	Custom (survey)
	Rancourt 2019	n=818 (F=475)	13-35yo PWT1D>1y, recruited via email through T1D Exchange Clinic Network – 70+ diabetes clinics across USA	Current	15.0% overall	DEPS-R (survey)
	Rydall et al. 1997	n=91	Adolescent females 12-18yo with T1D under care at the Hospital for Sick Children, Toronto	Current (past 3 m)	34% overall	DSED adaptation (survey)
	Schwartz et al. 2003	n=53	Female adolescents 12-18yo with T1D>1y recruited from diabetes clinics and satellite clinics in Illinois and Georgia, USA	Lifetime	17%	Custom, pilot tested (survey)

* akshelford@gmail.com

School of Psychology and Clinical Language Sciences, University of Reading, UK

Behaviour	Citation	n (Female)	Sample description	Point of prevalence	Prevalence [confidence intervals]	Measure (type)
	Stancin et al., 1989	n=59	Females 18-30yo with T1D from northeastern Ohio, USA	Current	39.0% overall	DSM-III bulimia screening; EDI (survey)
	Streigel-moore et al. 1992	n=46	Females with T1D>1y age 8-18yo on case register for Yale Children's Diabetes Center	Current (past 4 w)	6.5% overall	EDE adaptation (interview)
	Troncone et al. 2020a	n=200 (F=98)	Adolescents 13-18yo with T1D>1y attending diabetes clinic in south Italy with a caregiver	Current	61.2% F, 61.7% M partial<br>20.4% F, 22.5% M total	DEPS-R (survey)
	Troncone et al. 2023	n=1,562 (F=750)	DiabEaT1 project in Italy, adolescents 11-19yo with T1D>1y attending 1 of 30 outpatient diabetes clinics with a caregiver in North, central or Southern Italy	Current	42.4% F, 42.5% M overall	DEPS-R (survey)
	Watt et al. 2022	n=199 (F=100)	Adults with T1D aged 18-65yo under care of ambulatory clinics provided by a large tertiary hospital in Melbourne, Australia	Current	25.8% partial<br>8.1% total	DEPS-R (survey)
	Wisting 2013	n=770 (F=390)	Children 11-16yo with T1D listed in the Norwegian Childhood Diabetes Registry (all paediatric departments in Norway)	Current	36.8% F, 4.5% M partial<br>26.2% F, 9.4% M total	DEPS-R (survey)
	Yafei et al. 2023	n=265 (F=163)	Saudi PWT1D >1y 12-25yo recruited randomly from Jazan Endocrinology and Diabetes Center registry	Current	29.4% F, 24.5% M partial<br>14.1% F, 8.8% M total	DEPS-R (survey)

NOTE: AHEAD = Action for Health in Diabetes; BIS = Body Image Scale; DEPS-R = Diabetes Eating Problem Survey – Revised; DSED = Diagnostic Survey for Eating Disorders; DSM = Diagnostic and Statistical Manual; DSMP = Diabetes Self-Management Profile; EAT = Eating and Activity over Time; EDE-Q = Eating Disorder Examination – Questionnaire; EDI = Eating Disorder Inventory; EMA = Ecological Momentary Assessment; FSE = Fragebogen zur Symptomdiagnose von Eßstörungen (Questionnaire for the Diagnosis Of Eating Disorders); MY-Q = MIND (Monitoring Individual Needs in Diabetes) Youth Questionnaire; PWT1D = People with type 1 diabetes. F = female, M = male. Prevalence reporting as percentage of sample, "partial" indicates partial restriction of insulin dose, "total" indicates restriction of total insulin dose.

* akshelford@gmail.com

School of Psychology and Clinical Language Sciences, University of Reading, UK
